# Investigation of factors controlling PM_2.5_ variability across the South Korean Peninsula during KORUS-AQ

**DOI:** 10.1525/elementa.424

**Published:** 2020

**Authors:** Carolyn E. Jordan, James H. Crawford, Andreas J. Beyersdorf, Thomas F. Eck, Hannah S. Halliday, Benjamin A. Nault, Lim-Seok Chang, JinSoo Park, Rokjin Park, Gangwoong Lee, Hwajin Kim, Jun-young Ahn, Seogju Cho, Hye Jung Shin, Jae Hong Lee, Jinsang Jung, Deug-Soo Kim, Meehye Lee, Taehyoung Lee, Andrew Whitehill, James Szykman, Melinda K. Schueneman, Pedro Campuzano-Jost, Jose L. Jimenez, Joshua P. DiGangi, Glenn S. Diskin, Bruce E. Anderson, Richard H. Moore, Luke D. Ziemba, Marta A. Fenn, Johnathan W. Hair, Ralph E. Kuehn, Robert E. Holz, Gao Chen, Katherine Travis, Michael Shook, David A. Peterson, Kara D. Lamb, Joshua P. Schwarz

**Affiliations:** * National Institute of Aerospace, Hampton, Virginia, US; † NASA Langley Research Center, Hampton, Virginia, US; ‡ California State University, San Bernardino, California, US; § NASA Goddard Space Flight Center, Greenbelt, Maryland, US; ∥ Universities Space Research Association, Columbia, Maryland, US; ¶ EPA, Research Triangle Park, North Carolina, US; ** Department of Chemistry, University of Colorado, Boulder, Colorado, US; †† Cooperative Institute for Research in the Environmental Sciences, University of Colorado, Boulder, Colorado, US; ‡‡ National Institute of Environmental Research, Air Quality Research Division, Incheon, KR; §§ School of Earth and Environmental Sciences, Seoul National University, Seoul, KR; ∥∥ Hankuk University of Foreign Studies, Seoul, KR; ¶¶ Center for Environment, Health and Welfare Research, Korea Institute of Science and Technology, Seoul, KR; *** Department of Energy and Environmental Engineering, University of Science and Technology, Daejeon, KR; ††† Seoul Metropolitan Government Research Institute of Public Health and Environment, Gyeonggi-do, KR; ‡‡‡ Harim Engineering, Inc., Seoul, KR; §§§ Center for Gas Analysis, Korea Research Institute of Standards and Science, Daejeon, KR; ∥∥∥ Department of Environmental Engineering, Kunsan National University, Gunsan, KR; ¶¶¶ Department of Earth and Environmental Sciences, Korea University, Seoul, KR; **** US EPA/Office of Research and Development/Center for Environmental Measurement and Modeling, Research Triangle Park, North Carolina, US; †††† Science Systems and Applications Inc., Hampton, Virginia, US; ‡‡‡‡ Space Sciences Engineering Center, University of Wisconsin, Madison, Wisconsin, US; §§§§ U.S. Naval Research Laboratory, Monterey, California, US; ∥∥∥∥ NOAA Earth System Research Laboratory, Chemical Sciences Division, Boulder, Colorado, US

**Keywords:** PM_2.5_, Aerosols, Air quality, South Korea, KORUS-AQ

## Abstract

The Korea – United States Air Quality Study (May – June 2016) deployed instrumented aircraft and ground-based measurements to elucidate causes of poor air quality related to high ozone and aerosol concentrations in South Korea. This work synthesizes data pertaining to aerosols (specifically, particulate matter with aerodynamic diameters <2.5 micrometers, PM_2.5_) and conditions leading to violations of South Korean air quality standards (24-hr mean PM_2.5_ < 35 μg m^−3^). PM_2.5_ variability from AirKorea monitors across South Korea is evaluated. Detailed data from the Seoul vicinity are used to interpret factors that contribute to elevated PM_2.5_. The interplay between meteorology and surface aerosols, contrasting synoptic-scale behavior vs. local influences, is presented. Transboundary transport from upwind sources, vertical mixing and containment of aerosols, and local production of secondary aerosols are discussed. Two meteorological periods are probed for drivers of elevated PM_2.5_. Clear, dry conditions, with limited transport (Stagnant period), promoted photochemical production of secondary organic aerosol from locally emitted precursors. Cloudy humid conditions fostered rapid heterogeneous secondary inorganic aerosol production from local and transported emissions (Transport/Haze period), likely driven by a positive feedback mechanism where water uptake by aerosols increased gas-to-particle partitioning that increased water uptake. Further, clouds reduced solar insolation, suppressing mixing, exacerbating PM_2.5_ accumulation in a shallow boundary layer. The combination of factors contributing to enhanced PM_2.5_ is challenging to model, complicating quantification of contributions to PM_2.5_ from local versus upwind precursors and production. We recommend co-locating additional continuous measurements at a few AirKorea sites across South Korea to help resolve this and other outstanding questions: carbon monoxide/carbon dioxide (transboundary transport tracer), boundary layer height (surface PM_2.5_ mixing depth), and aerosol composition with aerosol liquid water (meteorologically-dependent secondary production). These data would aid future research to refine emissions targets to further improve South Korean PM_2.5_ air quality.

## 1. Introduction

The joint Korea – United States Air Quality Study (KORUS-AQ) conducted in May–June 2016 was tasked with clarifying the causes of poor air quality in South Korea, which is principally associated with high ozone (O_3_) and aerosol concentrations (Crawford et al., 2020). In this work, we focus on the aerosol influence by providing a synthesis of PM_2.5_ (particulate matter with aerodynamic diameter ≤2.5 μm) observed across the national AirKorea monitoring network, along with more detailed aerosol composition data in the vicinity of Seoul from ground-based monitoring stations and NASA’s DC-8 airborne platform. Over the course of the study period from May 1^st^ through June 10^th^, exceedances of the daily-average PM_2.5_ air quality standard of 50 μg m^−3^ were limited primarily to the last week of May (Rapid Science Synthesis Report, 2017; Crawford et al., 2020). Recent lowering of the standard in South Korea to 35 μg m^−3^ would bring some portion of the AirKorea network into noncompliance on a daily basis. Aerosol composition measurements show secondary species account for ~75% of PM_2.5_ (Rapid Science Synthesis Report, 2017), highlighting the need to understand the chemical and physical influences on PM_2.5_ variability.

The KORUS-AQ observations provide a rich dataset for interpreting the factors contributing to PM_2.5_ variability. This includes detailed trace gas and aerosol composition, both on the ground and from aircraft, and meteorological data at local and synoptic scales (Crawford et al., 2020; Peterson et al., 2019). These focused observations allow for an exploration of the following factors influencing PM_2.5_ at the surface and aerosol properties throughout the boundary layer:

1. Transboundary transport of aerosol pollution from upwind sources
2. Changes in vertical mixing and containment of local aerosol pollution
3. Local processes that result in secondary production of aerosol

Each of these processes are subject to the influence of meteorological parameters, such as solar radiation; surface and air temperatures; relative humidity; the presence of haze, fog, and clouds; diurnal variations of the boundary layer depth; and horizontal transport regimes, whether long-range (i.e., transboundary) or local (e.g., land-sea breezes). Analysis of the KORUS-AQ observations is aided by the occurrence of four distinct synoptic meteorological periods during the study. These periods are discussed in more detail by Peterson et al. (2019) but are summarized here as follows:

*Dynamic period* (1–16 May), characterized by a succession of frontal passages that limited the accumulation of aerosol at the surface.

*Stagnant period* (17–22 May), during which dry conditions under a persistent anticyclone led to clear skies, a wide diurnal temperature range, and limited transport into South Korea from external sources, such that local emissions and atmospheric processes dominated surface observations of atmospheric constituents.

*Transport/Haze period* (25–31 May), which included four frontal passages, weaker than those during the dynamic period, that brought polluted air from China to the Korean peninsula, but with more limited vertical motion, which focused horizontal transport near the surface. Each front was associated with extensive cloud cover, and high humidity, which promoted low visibility due to haze and fog development within a shallow boundary layer.

*Blocking period* (1–7 June), during which a Rex Block (Peterson et al., 2019, and references therein) limited transport, resulting in occasional stagnant conditions that did not persist long enough to accumulate surface pollutants to the extent observed during the Stagnant period.

Previous analyses of aerosols during KORUS-AQ have been more narrowly focused on a specific site (Kim et al., 2018) or component of aerosol composition such as organic aerosol (Nault et al., 2018) or black carbon (Lamb et al., 2018). This work builds on these previous studies by offering a more comprehensive evaluation of aerosol observations and supporting data from the breadth of airborne and ground-based observations, including regulatory monitors, collected during the study period. This broader analysis is of particular importance for understanding shortfalls in previous studies showing that despite the ability of large-scale models to reproduce the general behavior of PM_2.5_ in South Korea during the KORUS-AQ period, models still fail to capture peak PM_2.5_ mass loadings and variability in aerosol composition (Choi et al., 2019). Using the GEOS-Chem model Choi et al. were unable to reproduce either the strong secondary organic aerosol (SOA) production that resulted in PM_2.5_ exceeding the mandated 24-hr average of 35 μg m^−3^ during the Stagnant period, or the combined increases of organic and inorganic aerosol components that exceed that standard during the Transport/Haze period. The limitations in reproducing SOA production during the Stagnant period were discussed in detail by Choi et al. and are related to incomplete knowledge of the photochemical pathways from which SOA is produced from myriad local gas-phase volatile organic compound (VOC) precursors under the clear skies and stagnant conditions of this period. This is further explored by Nault et al. (2018) in an analysis of KORUS-AQ aerosol observations from the NASA DC-8 and is also consistent with previous studies (e.g., Heald et al., 2010; Hodzic et al., 2016, 2020; Schroder et al., 2018; Woody et al., 2016). The underprediction of observed aerosol concentrations during the Transport/Haze period has not been explored in detail, but a combination of factors has been suggested for the aerosol observations involving both transport and enhanced local aerosol production under the cloudy moist conditions during this period (Kim et al., 2018; Peterson et al., 2019).

Given the charge to the KORUS-AQ team to assess poor air quality related to aerosols and offer guidance that can help policy makers in South Korea take steps to improve air quality, we present aerosol observations obtained during the KORUS-AQ campaign, with a focus on the contrast between the two meteorological periods when persistent high PM_2.5_ mass loadings were the most acute: Stagnant and Transport/Haze (Peterson et al., 2019). We begin with an examination of the peninsula-wide PM_2.5_ observations from the ground-based AirKorea network to broadly characterize the role of synoptic meteorology on PM_2.5_. Then, we supplement those observations with more detailed data sets from Seoul obtained at ground sites and from vertical profiles over the city by the DC-8. The contrast in the meteorological conditions between the Stagnant and Transport/Haze periods are used to highlight different pathways that lead to elevated PM_2.5_ in South Korea. A short springtime field campaign is insufficient to resolve all of the outstanding questions related to poor air quality due to aerosols and the various factors involved in enhanced PM_2.5_ throughout the year. Nevertheless, the density and breadth of observations collected during KORUS-AQ and the observed gradients in fine particle pollution with changes in meteorological conditions serve to advance understanding. Specifically, the data obtained during KORUS-AQ and its analysis sheds light on priorities for routine data collection at a subset of AirKorea network sites that hold the greatest promise for resolving outstanding uncertainties.

## 2. Methods

A brief synopsis of the data and calculations employed in this work is provided here, with the reader encouraged to see the accompanying references for further details. All data are available from the KORUS-AQ data archive via the digital object identifier (doi) 10.5067/Suborbital/KORUS-AQ/DATA01.

### 2.1. Data sources

#### 2.1.1. AirKorea PM_2.5_ data

The AirKorea network is operated by South Korea’s National Institute of Environmental Research (NIER). Hourly PM_2.5_ mass loadings (μg m^−3^) were measured with BAM-1020 instruments (Met One Instruments, Inc., Grants Pass, OR, USA) that use beta-ray attenuation (Choi et al., 2019).

#### 2.1.2. Korea Institute of Science and Technology (KIST) data

Sampling was conducted on the 5^th^ floor of a building on the KIST campus (37.6015°N, 127.0452°E) about 11 km northwest of Olympic Park, north of the Han River (Kim et al., 2018). An Aerodyne Research, Inc., (Billerica, MA, USA) high resolution time-of-flight aerosol mass spectrometer (HR-ToF-AMS) (DeCarlo et al., 2006) was used to measure non-refractory PM_1_ (particulate matter with aerodynamic diameter ≤1 μm) composition (nitrate, sulfate, ammonium, chloride, and organic aerosol), while a Thermo Fischer Scientific (Waltham, MA, USA) multi-angle absorption photometer (MAAP) was used to measure black carbon (BC) (Kim et al., 2017, 2018). The inflow to these instruments passed through a PM_2.5_ cyclone (URG Corp., Chapel Hill, NC, USA) and a Nafion dryer (PermaPure LLC, Lakewood, NJ, USA). Meteorological data (from the Korea Meteorological Administration) to accompany the KIST measurements were obtained from the nearby Jungreung site (37.61°N, 127.00°E). For details see Kim et al. (2017, 2018).

#### 2.1.3. Olympic Park data

The Olympic Park site was operated by the Seoul Metropolitan Government Research Institute of Public Health and Environment during KORUS-AQ (Choi et al., 2019). Located in the middle of Seoul on the south side of the Han River (37.5216°N, 127.1242°E; 26 m above sea level), the site was set up in a temporary shelter outside the Seoul Historiography Institute building. Measurements commenced May 8^th^, a week after the start of the airborne campaign. A Teledyne API T640 light spectrometer (Teledyne API, Inc., San Diego, CA, USA) was used to measure PM_2.5_ at a comparable time resolution (5 min) to the AMS (Aerodyne Research, Inc., Billerica, MA, USA) measurement at this site. Hourly measurements of temperature (T) and relative humidity (RH) were made with a Met One 083D-1–35 instrument (Met One Instruments, Inc., USA). Hourly solar radiation measurements were made with Met One 096–1 (Met One Instruments, Inc., USA) instrument. An Ecotech (Melbourne, Australia) model EC9841 was used to measure hourly nitric oxide (NO) and nitrogen dioxide (NO_2_), while model EC9810 was used to measure O_3_. Hourly nitric acid (HNO_3_) was measured using two methods: a MARGA ADI 2080 instrument (Doga, Ltd., Ankara, Turkey), along with a custom high efficiency denuder scrubber (HEDS) coupled to two ion chromatograph (IC) systems (Song et al., 2009). Aerosol backscatter profiles were measured at 910 nm by a CL51 ceilometer (Vaisala Corp., Helsinki, Finland) set up about 4 m above the ground. Mixing layer heights (MLH) were derived from gradients in aerosol backscatter profiles by the Vaisala BLView software (Knepp et al., 2017).

#### 2.1.4. DC-8 data

Aerosol optical properties for particles up to 5 μm in diameter were measured by a suite of instruments including TSI Inc. model 3563 nephelometers (Shoreview, MN, USA) for aerosol scattering, a Radiance Research particle soot absorption photometer (Seattle, WA, USA) for absorption, and a Laser Aerosol Spectrometer (LAS, model 3340, TSI, Inc., Shoreview, MN, USA) for aerosol size distribution between 100 nm and 5 μm. Speciated non-refractory PM_1_ was sampled with a highly customized (University of Colorado-Boulder) Aerodyne Research, Inc. (Billerica, MA, USA) HR-ToF-AMS (DeCarlo et al., 2006). Details of this HR-ToF-AMS for the KORUS-AQ campaign can be found in Nault et al. (2018). Refractory BC concentrations, size distributions, and microphysical state in the accumulation mode were measured with a single particle soot photometer (SP2, Droplet Measurement Technologies, Longmont, CO, USA) (Lamb et al., 2018). A modified LI-COR 6252 (LI-COR, Inc., Lincoln, NE, USA) instrument (Vay et al., 2003) was used to measure CO_2_ and the NASA DACOM instrument (Sachse et al., 1987; Sachse et al., 1991) was used to measured CO, see Halliday et al. (2019) for details. OH was measured by Laser Induced Fluorescence (Faloona et al., 2004). RH was calculated based on measurements of water vapor concentration by an open-path diode laser hygrometer (Diskin et al., 2002) coupled with static temperature and pressure data provided by the DC-8 meteorological measurement system.

### 2.2. Calculation to estimate PM_1_ Aerosol Liquid Water (ALW)

For Olympic Park and KIST, the thermodynamic Extended Aerosol Inorganic Model (E-AIM) Model II (Clegg et al., 1998; Massucci et al., 1999; Wexler and Clegg, 2002) was used to calculate ALW mass concentration for PM_1_ aerosol. Inputs into the model were aerosol nitrate, sulfate, ammonium, gas-phase HNO_3_, RH, and T. ALW from Olympic Park includes HNO_3_ in the calculation; however, HNO_3_ was not measured at KIST. A sensitivity analysis found there was no difference in ALW whether HNO_3_ was included or not in the thermodynamic model for Olympic Park, suggesting the calculation at KIST was acceptable. Model II only considers the inputs listed above, and not aerosol chloride, providing a wider RH and T range to calculate ALW (Friese and Ebel, 2010). The ALW calculated with E-AIM generally agrees well with that from other aerosol thermodynamic models (Metzger et al., 2016).

### 2.3. Estimation of the nitrate (NO_3_) radical and dinitrogen pentoxide (N_2_O_5_) at Olympic Park

Estimation of the NO_3_ radical and N_2_O_5_ abundance relies on measurements of NO, NO_2_, O_3_, alkenes, aerosol surface area, and N_2_O_5_ uptake. At Olympic Park there were only measurements of NO, NO_2_, and O_3_, which greatly limits the ability to determine NO_3_ radical and N_2_O_5_ steady state concentrations. Continual fresh emissions of NO and alkenes further complicate calculations, as NO_3_ radicals may never reach steady state at Olympic Park. Additionally, high mixing ratios of NO_2_ also limit the ability for NO_3_ radicals and N_2_O_5_ to reach steady state (Brown et al., 2003). Nonetheless, the production of NO_3_ radicals (Eq. 1), relative losses of NO_3_ radicals (Eqs. 2 and 3), and N_2_O_5_ heterogeneous uptake rate constant (Eq. 4) can be estimated.

(Eq. 1) $$P\left(NO_{3}\right)=k\left[NO_{2}\right]\left[O_{3}\right]$$

(Eq. 2) $$L{\left(NO_{3}\right)}_{NO}=k[NO]$$

(Eq. 3) $$L{\left(NO_{3}\right)}_{NO_{2}}=K\left[NO_{2}\right]$$

(Eq. 4) $$k_{N_{2}O_{5}}=\frac{1}{4}\bar{c}\gamma \left(N_{2}O_{5}\right)S_{A}$$

Here, all rate constants related to the NO_3_ radical (Eqs. 1–3) were obtained from Burkholder et al. (2015). For the heterogeneous uptake of N_2_O_5_ to aerosol (Eq. 4), S_A_ is the surface area of aerosol (calculated based on the LAS size distribution, see Section 2.1.4, assuming spherical aerosols), with mean values from the DC-8 data set used here, as S_A_ was limited to <250 nm at the ground site. γ is the N_2_O_5_ uptake, calculated using the parameterization from Bertram and Thornton (2009), along with the measured chloride and nitrate from the Olympic Park AMS and calculated ALW (Section 2.2). Finally, c̅ is the mean molecular speed of N_2_O_5_ determined from gas kinetic theory and the observed ambient T.

## 3. Observations

### 3.1. Overview of PM_2.5_ behavior during KORUS-AQ

**Figure 1:**
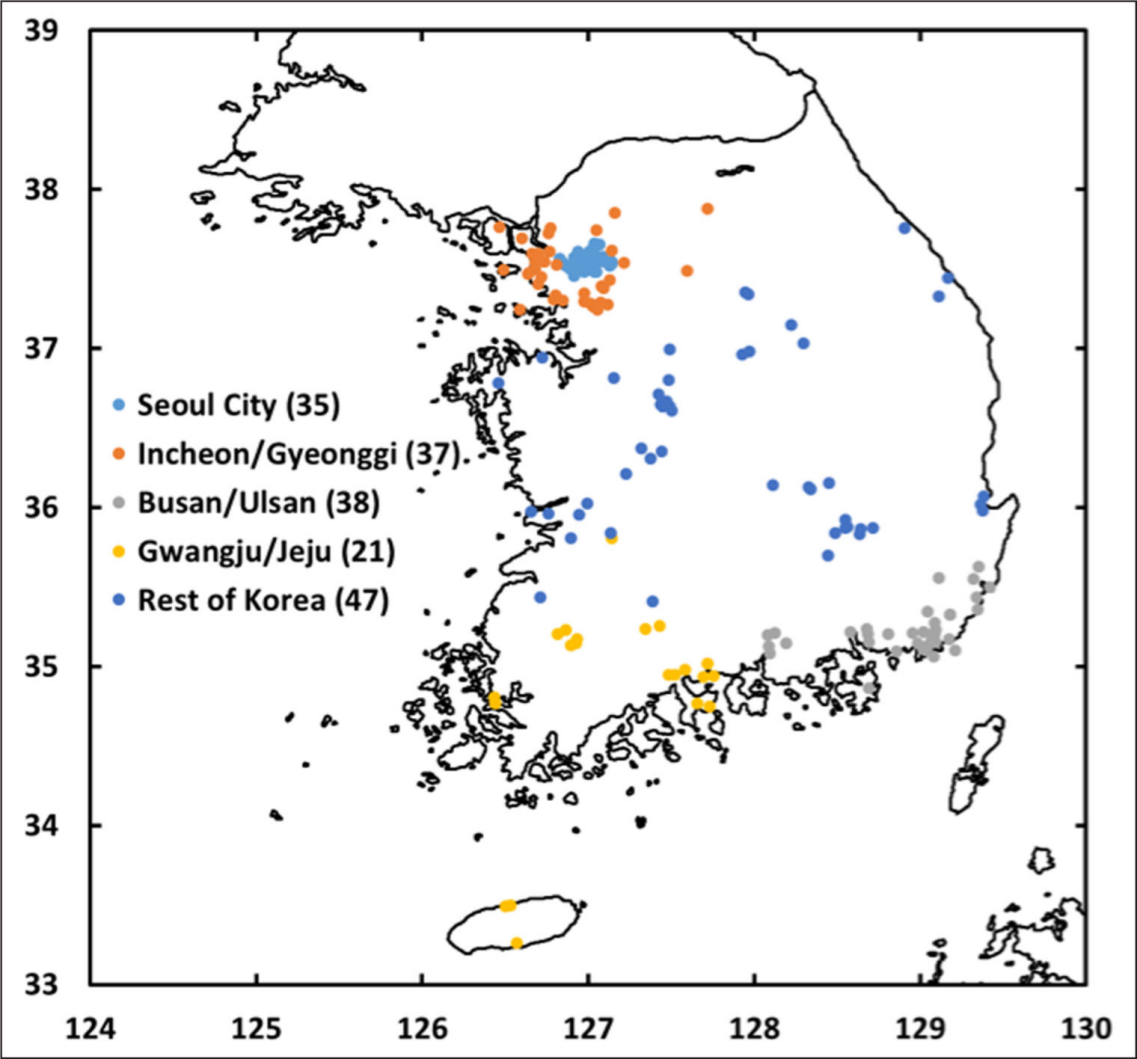
Map of AirKorea sites divided into five sectors. The Seoul Metropolitan Area (SMA) encompasses Seoul City, Incheon, and Gyeonggi. Southeastern coastal sites include Busan and Ulsan. Southwestern sites include Gwangju and Jeju island. Sites across central S. Korea are referred to as “Rest of Korea”. The number of sites in each sector are provided in parentheses. DOI: https://doi.org/10.1525/elementa.424.f1

**Figure 2:**
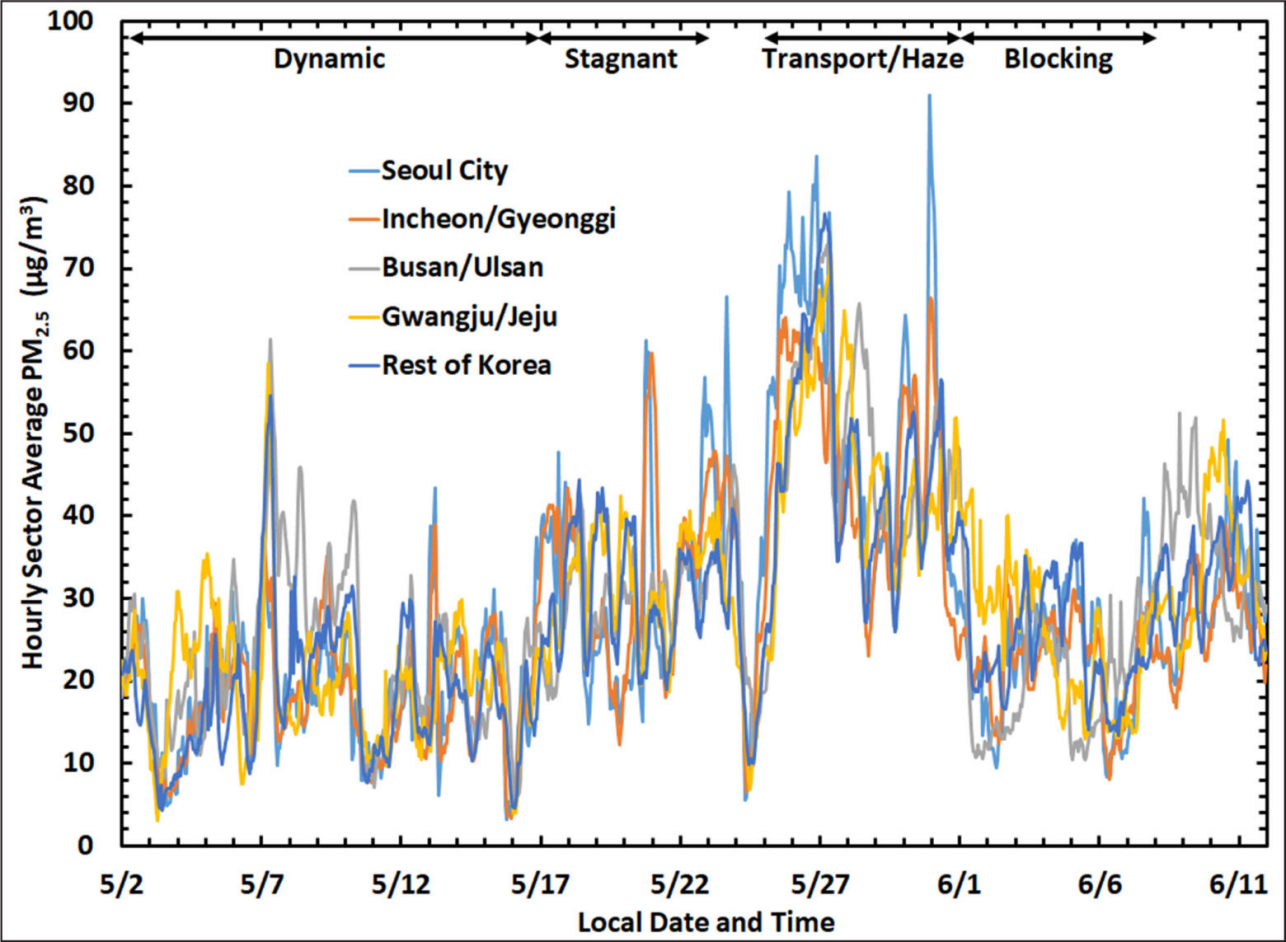
Time series of hourly PM_2.5_ concentrations averaged across each of the five sectors shown in Figure 1. The four major meteorological periods identified in Peterson et al. [2019] are annotated across the top of the figure. DOI: https://doi.org/10.1525/elementa.424.f2

South Korea has an extensive network of air monitoring sites (AirKorea, Figure 1) that record PM_2.5_ (see Section 2.1.1). To assess the variability of PM_2.5_ across the peninsula, the sites were grouped into five geographic sectors (Figure 1) with the mean hourly PM_2.5_ calculated for each sector during the KORUS-AQ campaign (Figure 2). Sites in the city of Seoul were separated from the surrounding provinces of Incheon and Gyeonggi, which together encompass the greater Seoul Metropolitan Area (SMA). The southeastern portion of the peninsula included sites in the cities of Busan and Ulsan and surrounding areas. The southwest sector included the city of Gwangju as well as Jeju island. The remaining sites across the central part of the peninsula were designated as the Rest of Korea.

The time series of hourly average PM_2.5_ for these sectors is shown in Figure 2, with the four major synoptic periods from Peterson et al. (2019) annotated across the top of the figure. There are several noteworthy features in the time series, the first of which is the similarity in the large-scale temporal variability across each of the sectors. This similarity in large-scale temporal variability can be quantified by comparing the daily-average PM_2.5_ at each site with the daily-average PM_2.5_ across the entire AirKorea network. The regression in Figure S1 shows that about two-thirds of the variability (r^2^ = 0.64) in PM_2.5_ at individual sites can be explained by the daily average across the peninsula. A second regression in Figure S2 examines whether there are any persistent spatial differences by comparing the daily-average PM_2.5_ values at each site against its own long-term average value during the KORUS-AQ study period. The low correlation (r^2^ = 0.13) indicates that there are no sectors that consistently experience higher PM_2.5_ than the rest of the peninsula. This is emphasized in Figure S2 by highlighting the data for the sites in Seoul which do not exhibit any tendency toward higher average PM_2.5_ values. Average sector values of PM_2.5_ for the entire study period differed by less than 1 μg m^−3^ from the peninsula-wide average of 28.4 μg m^−3^. These comparisons indicate that the daily variability in PM_2.5_ across the AirKorea network depended less on location than on temporal changes in synoptic meteorology.

An example of synoptic influence across the Korean peninsula is seen in the minimum PM_2.5_ levels observed for all sectors due to removal during precipitation events on May 3^rd^, 16^th^, and 24^th^ (Figure 2). Other meteorological factors affecting PM_2.5_ include transboundary transport (see Section 3.2.1), land/sea breeze circulations (see Sections 3.2.1 and 3.2.3), boundary layer depth (see Section 3.2.2), T, and RH (see Section 3.2.4). These factors contribute not only to the coherence of PM_2.5_ across the peninsula, but to the differences observed among the sectors as well, since a particular transport pathway, the extent of cloud cover, fog occurrence, or other phenomena may not encompass the entire country. For example, Gwangju/Jeju sites exhibited elevated PM_2.5_ compared to the other sectors on May 4^th^–5^th^ (Dynamic period) and June 1^st^–3^rd^ (Blocking period), whereas Busan/Ulsan sites were elevated on May 8^th^–10^th^ (Dynamic period) and June 8^th^–9^th^ (Figure 2). The SMA tended to have higher PM_2.5_ than the rest of the peninsula when PM_2.5_ exceeded 50 μg m^−3^, which occurred several times between May 20^th^ and June 1^st^ (Stagnant and Transport/Haze periods, Figure 2).

Given the focus on better understanding of the drivers of poor air quality related to aerosol, the remainder of this work investigates the factors contributing to the highest PM_2.5_ mass loadings, which were more prevalent during the Stagnant and Transport/Haze periods (Figure 2), with the highest concentrations occurring in the SMA. These two periods will be contrasted in terms of meteorological conditions and aerosol observations from the AirKorea network and will take advantage of the greater information available for the SMA from research-grade measurements at the KORUS-AQ ground sites and onboard the NASA DC-8 aircraft.

### 3.2. Evidence for drivers of PM_2.5_ pollution episodes

#### 3.2.1. Transboundary transport

**Figure 3:**
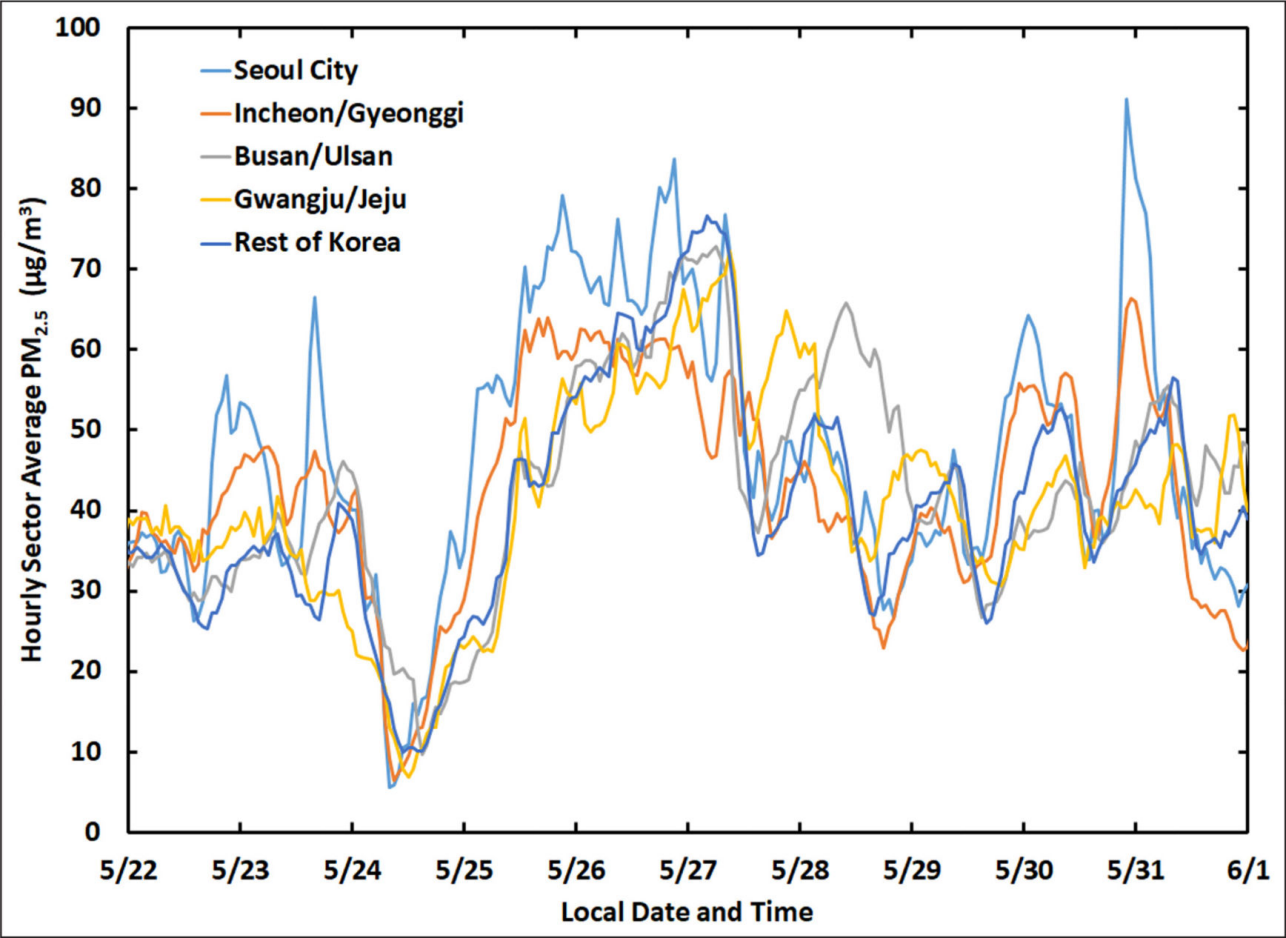
Subset of the time series in Figure 2 focusing on the Transport/Haze period. DOI: https://doi.org/10.1525/elementa.424.f3

The highest PM_2.5_ concentrations occurred during the Transport/Haze period (Figure 2) with a mean (±1σ standard deviation) of 47.9 ± 0.1 μg m^−3^ for the entire AirKorea network over that period. In contrast, the network mean for the Stagnant period was 30.7 ± 0.1 μg m^−3^. The difference of 17 μg m^−3^ is substantial, and this difference nearly doubled to 32 μg m^−3^ for the PM_2.5_ peak observed on May 26^th^. The difference in mean PM_2.5_ between these two periods might be considered to represent only the additional contribution from upwind sources, given the limited transboundary transport of the Stagnant period versus the weak frontal passages of the Transport/Haze period bringing aerosols into the region from China described by Peterson et al. (2019). However, the role of transport alone is challenged when focusing on the details of the time series of hourly sector means of PM_2.5_ during the Transport/Haze period shown in Figure 3. PM_2.5_ rapidly increased across the peninsula following a rain-induced minimum of less than 10 μg m^−3^ on May 24^th^. The increase occurred much more quickly in Seoul and surrounding provinces than across the rest of the peninsula. Seoul City PM_2.5_ increased faster and reached concentrations that were ~20 μg m^−3^ greater than Incheon/Gyeonggi during the initial increases on May 25^th^–27^th^ and again on May 31^st^ (Figure 3). If the changes in PM_2.5_ were solely due to transboundary transport, increases across the SMA would be expected to be similar. The substantially higher PM_2.5_ in Seoul versus Incheon/Gyeonggi deserve further scrutiny.

**Figure 4:**
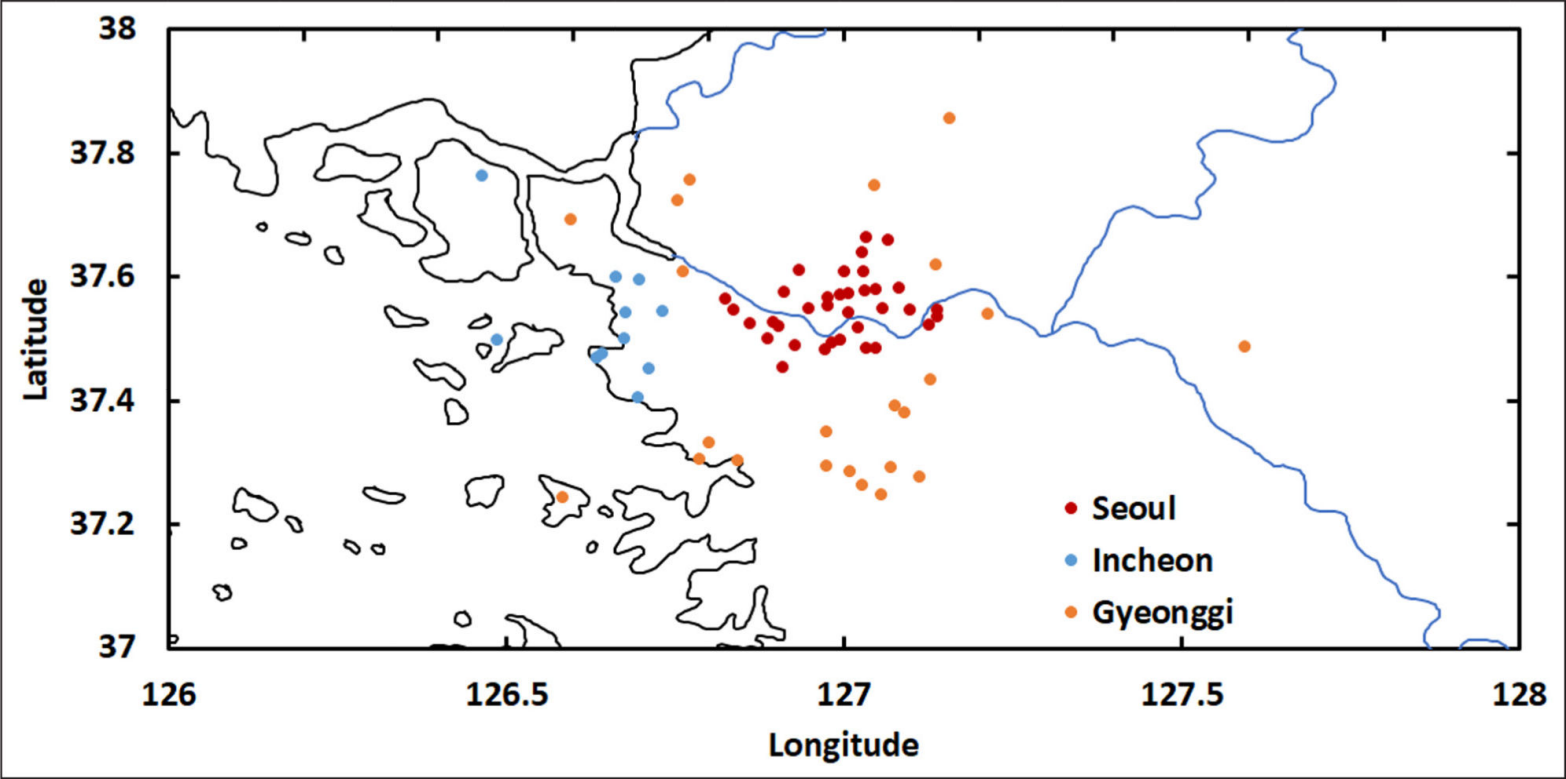
Locations of AirKorea sites in the SMA divided into three sectors: Seoul City, Incheon, and Gyeonggi. DOI: https://doi.org/10.1525/elementa.424.f4

**Figure 5:**
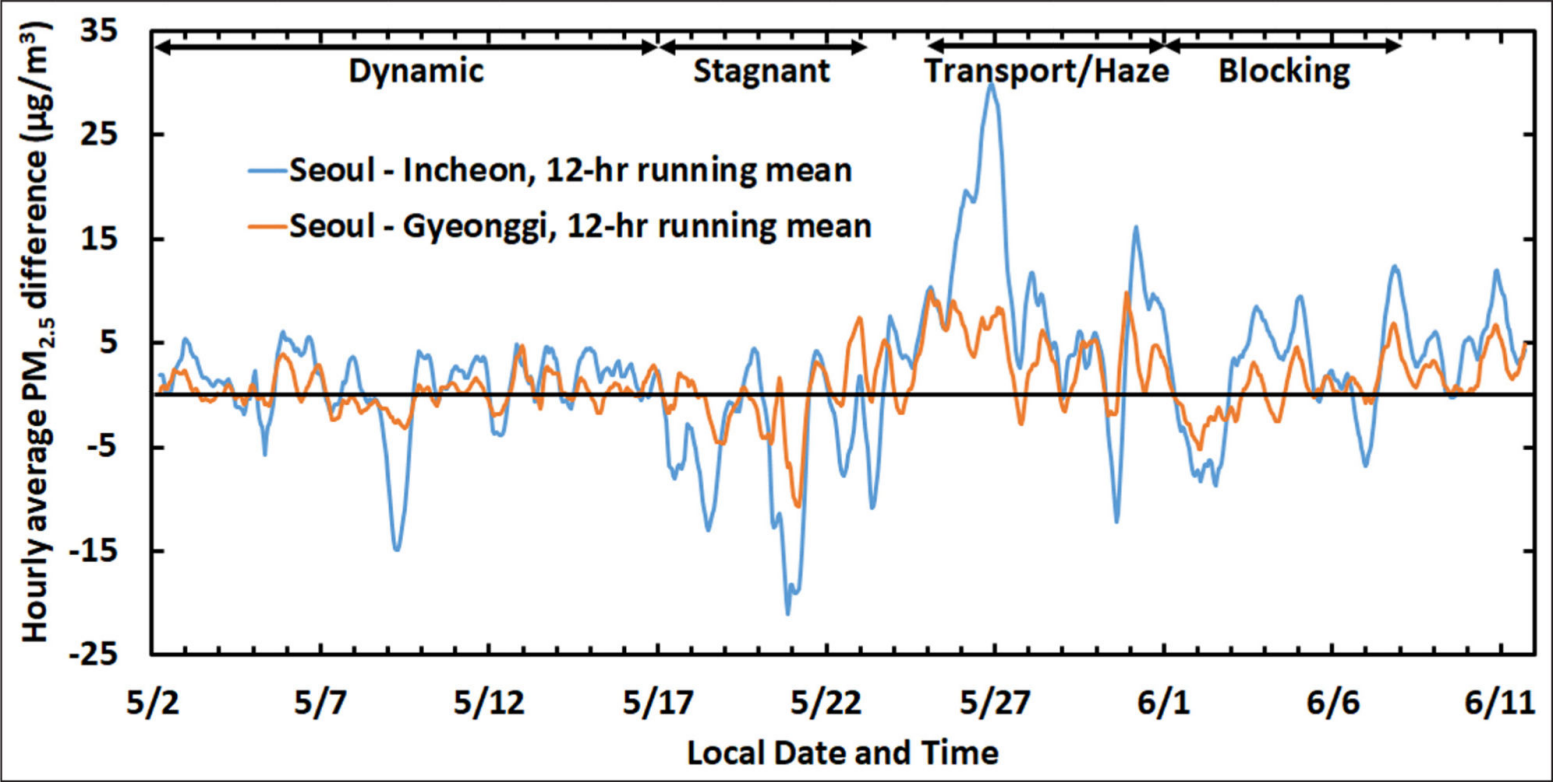
Time series of the difference between average PM_2.5_ concentrations for AirKorea sites in Seoul City and sites in Incheon and Gyeonggi. A 12-hr running mean has been applied to the hourly differences. As in Figure 2, meteorological periods are annotated at the top of the figure. DOI: https://doi.org/10.1525/elementa.424.f5

Figure 4 provides greater detail on AirKorea sites in the SMA by separating the Incheon and Gyeonggi sites. The Incheon sites occupied the coastal zone to the west of Seoul, while Gyeonggi sites occupied an arc around the city to the north, east, and south. Differences in PM_2.5_ across the SMA are quantified in Figure 5 by taking the difference between the Seoul sector PM_2.5_ and the surrounding provinces of Incheon and Gyeonggi. Here, a 12-hr running mean of the hourly average PM_2.5_ is applied to more clearly show the differences between these sectors.

Sector differences in Figure 5 stand out during the Transport/Haze period with the difference between Seoul and Incheon PM_2.5_ averaging 9.6 ± 0.9 μg m^−3^ over the entire period. The largest daily mean difference between these sectors was observed on May 26^th^ with Seoul exceeding Incheon by 23.6 ± 1.7 μg m^−3^. With the Incheon sites positioned directly to the west of Seoul, this difference suggests that a substantial increment of PM_2.5_ in Seoul was not due to transport. The higher concentration of aerosol precursors in Seoul, especially for reactive nitrogen, points to the potential for local sources to drive the higher PM_2.5_ in Seoul during the Transport/Haze period. The difference with Gyeonggi was smaller but still significant with a mean enhancement of 4.2 ± 0.5 μg m^−3^ over the entire period.

Large negative differences also occur in the time series, particularly during the Stagnant period when PM_2.5_ in Incheon exceeded Seoul by 4.8 ± 0.9 ug/m^3^. The most significant difference during this period occurred on May 20^th^, when a substantial land/sea-breeze circulation transported pollution offshore early in the day (land breeze) and returned it inland by late afternoon (sea breeze). This event is described in detail by Peterson et al. (2019). It is expected that local land/sea-breeze influences would be more prevalent during the Stagnant period when synoptic-scale dynamics were weakest. Land/sea-breezes are indicative of the strong influence of local transport on PM_2.5_, even in the absence of influences from transboundary transport from upwind Asian sources. Under such conditions local emissions controls could reduce precursors carried west over the water where secondary products are formed in the marine boundary layer and hence, could help to reduce pollutants subsequently brought back onshore later in the day.

For the Dynamic and Blocking periods, differences were both smaller and more evenly distributed between positive and negative values, such that average differences were on the order of 1 μg m^−3^ or less. The lack of a clear tendency for these two periods provides an important contrast to the gradients across the SMA that characterized the Transport/Haze and Stagnant periods. A similar analysis using both PM_2.5_ and AERONET data supports this assessment (Eck et al., 2020).

**Figure 6:**
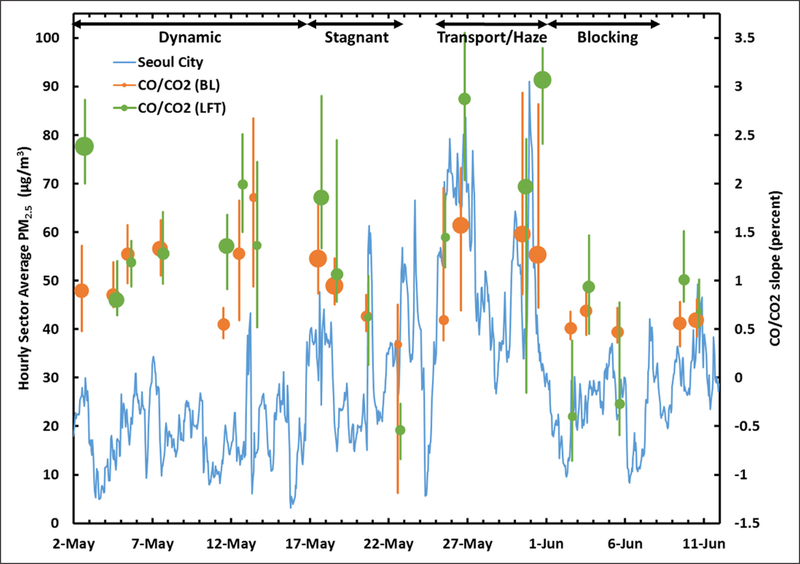
Time series of the hourly average PM_2.5_ concentrations during the KORUS-AQ period for AirKorea sites in Seoul City. Median CO/CO_2_ slopes are shown for DC-8 flight days and are taken from descent profiles over Seoul. Statistics for the CO/CO_2_ slopes (median and interquartile range) are shown for 1-minute data periods with slopes having a significant correlation (R^2^ > 0.5). Symbol size is proportional to the fraction of data having significant CO/CO_2_ correlation. Statistics are shown for both the boundary layer (BL, orange) and lower free troposphere (LFT, green). As in Figure 2, meteorological periods are annotated at the top of the figure. DOI: https://doi.org/10.1525/elementa.424.f6

While the above analysis suggests that local emissions and atmospheric processing played an important role in determining PM_2.5_ during the Transport/Haze period, it does not eliminate the expectation that the period was also influenced by transport from China. To further examine the role of transboundary transport during the KORUS-AQ study, a metric based on the covariance of CO and CO_2_ observations from the DC-8 is shown along with the time series of the hourly average PM_2.5_ of the Seoul City sector in Figure 6. Halliday et al. (2019) developed this metric by examining slopes for high-resolution (1 Hz) CO/CO_2_ measurements over a rolling one-minute period. This metric is useful for distinguishing sources of combustion in this region, since sources in China differ in their emissions profile from those in South Korea. Halliday et al. (2019) provides a detailed discussion of the evidence for these differences in emissions based on both published observations as well as concurrent airborne observations conducted in China’s Hebei province during the ARIAs (Air Chemistry Research in Asia) study (He et al., 2017) which are also archived with the KORUS-AQ data. To summarize the metric, higher slopes in CO/CO_2_ indicate inefficient combustion, with values as high as 4% in fresh emissions from China. Values closer to 1% are consistent with South Korean emissions. Negative values can also occur when air has experienced strong CO_2_ uptake due to biogenic activity. CO_2_ was not measured at the ground sites in Seoul, so a complete time series of this metric is not available, nonetheless, the DC-8 observations provide evidence of transboundary transport influencing Seoul throughout the KORUS-AQ study period.

Looking at this metric in Figure 6, there are several notable behaviors that are consistent with expectations for each meteorological period. The highest CO/CO_2_ slopes in the boundary layer (BL) occurred during the Dynamic and Transport/Haze periods when the potential for transboundary influence was the greatest. Median values during the Transport/Haze period were not much larger than during the initial Dynamic period, but the larger symbol sizes indicate a more robust relationship and the upper end of the interquartile range reached higher values during the Transport/Haze period, demonstrating the greatest transboundary influence during this time. Similarly, the statistics for the CO/CO_2_ metric in the lower free troposphere (LFT) also exhibited values during the Transport/Haze and Dynamic periods well above those observed at other times, consistent with the frontal lifting of pollution and expectation for greater transport overhead than at the surface. Vertical profiles of BC also showed higher loading of BC in the lower free troposphere than at the surface during the Transport/Haze period (Lamb et al., 2018).

During the Stagnant period, the initially high CO/CO_2_ slope decreased over time in both the BL and LFT, consistent with the gradual dilution of transboundary influence as contributions from local sources grew the longer the air mass over the South Korean peninsula was isolated from outside sources. Values varied little during the Blocking period, with similar values to the late Stagnant period, suggesting local sources were important at these times. While the CO/CO_2_ behavior is generally consistent with the expectation and timing of transboundary influences per Peterson et al. (2019), it is insufficient to quantify the magnitude of PM_2.5_ attributable to transport from sources outside South Korea. It also does not negate the questions raised by the differences between Seoul and the rest of SMA (Figure 5) that point to additional factors related to local emissions and processes contributing to PM_2.5_ variability.

#### 3.2.2. Boundary layer influences

**Figure 7:**
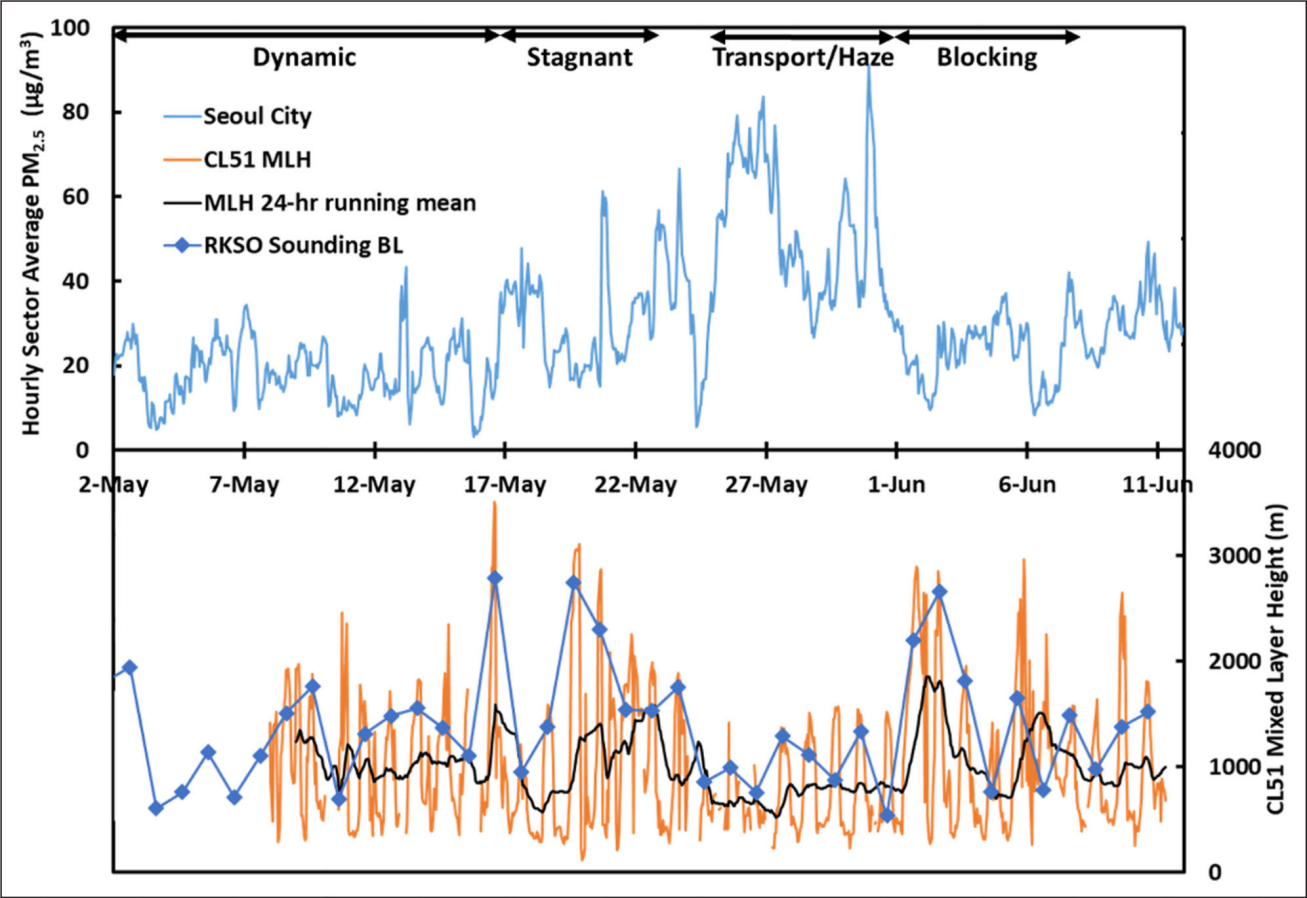
Time series of the hourly average PM_2.5_ during the KORUS-AQ period for AirKorea sites in Seoul (top panel). Time series of hourly average mixed layer height (MLH) measured at Olympic Park in Seoul by a CL51 ceilometer (bottom panel) along with a 24-hr running mean showing MLH over the previous 24 hrs. BL depth based on daily soundings in the afternoon (3 p.m. local time) at Osan Air Base are also shown in the bottom panel. As in Figure 2, meteorological periods are annotated at the top of the figure. DOI: https://doi.org/10.1525/elementa.424.f7

An examination of boundary layer height provides an indication of the degree to which local sources are contained, with lower heights leading to greater pollution experienced at the surface as emissions are mixed into a smaller volume. Here, the boundary layer height (typically described meteorologically) is characterized by a laser-based methodology (see Section 2.1.3) that detects the near-surface pollution layer using the observed aerosol gradient (Knepp et al., 2017). For clarity, the term mixed layer height (MLH) is used for this approach. MLH provides a useful metric related to the same particle pollution being measured by the surface PM_2.5_ monitors. The time series of both MLH and Seoul City PM_2.5_ are shown in Figure 7. Hourly average MLH values exhibit strong diurnal variability, with low values at night and higher values during the day, as expected. Less day-to-day variability is observed for nighttime MLH than is observed for daytime MLH throughout the KORUS-AQ study period. The Transport/Haze period in particular exhibits a sustained period of low daytime MLH values, highlighted using a 24-hour running mean. This suggests containment of local pollution within a shallow mixed layer throughout the day played a role in the observed increase in PM_2.5_ during the Transport/Haze period. For additional confidence, BL depth diagnosed from daily soundings at Osan Air Base at 3 p.m. local time are also shown in Figure 7. Not shown are CL51 MLH data collected concurrently at the Taehwa Forest research site that closely corroborates the Olympic Park ceilometer. The similarity between Olympic Park, Osan Air Base (50 km to the south), and Taehwa Forest (30 km to the southeast) confirm the coherence in boundary layer behavior across the SMA as it responded to meteorological conditions.

Direct evidence of containment cannot be derived from PM_2.5_, given that it is influenced by both primary emissions and secondary production. NO_x_ (the sum of NO and NO_2_) provides a much more effective measure as it has a strong local source emission with no appreciable secondary sources and a short lifetime to preclude large influences from transboundary transport. However, evidence for containment of NO_x_ is complicated by its diurnal variability (Fig. S3), as nighttime values depend heavily on available O_3_ levels to drive nighttime conversion of NO_x_ to HNO_3_ (see Section 4.2.1). As will be discussed later, the NO_x_ lifetime changes substantially at night throughout the KORUS-AQ period, with less nighttime NO_x_ during the Transport/Haze period (Fig. S3). Since the dynamics of the nocturnal boundary layer are not well represented by the aerosol gradients detected by the ceilometer, there is little information to discern subtle differences in nighttime mixing. Thus, the lack of O_3_ titration is one of the best indicators of a deeper nocturnal boundary layer. These events also coincide with cloud cover and warmer nighttime temperatures which may contribute to sustaining a deeper nocturnal boundary layer.

Focusing on afternoon conditions, NO_x_ values are greater during the Transport/Haze period than during other periods of the KORUS-AQ study. Afternoon is the time of greatest contrast as it represents the culmination of daytime boundary layer growth. Average afternoon NO_x_ (12:00–17:00 local time) during the Transport/Haze period at Olympic Park was 41.7 ± 1.5 ppbv, nearly 12 ppbv (or 40%) greater than observed during the rest of the KORUS-AQ period (29.9 ± 1.0 ppbv). This suggests that containment alone could have had a substantial influence on surface PM_2.5_, but it is also important to assess the effect of meteorological conditions on atmospheric processes involved in secondary aerosol formation from local sources (see Section 3.2.3 and Section 4).

The suppressed boundary layer growth during the Transport/Haze period resulted in reduced entrainment from the lower free troposphere. This is consistent with the larger differences in CO/CO_2_ slopes observed between the BL and LFT during the Transport/Haze period (Figure 6). While a full evaluation of the meteorological factors governing the changes in boundary layer depth are beyond the scope of this paper, the dominant factor driving the low values during the transport period was likely the heavier cloud cover during this period leading to observed reductions in surface insolation and less surface heating to drive mixing (Eck et al., 2020).

#### 3.2.3. Aerosol composition across meteorological periods

**Figure 8:**
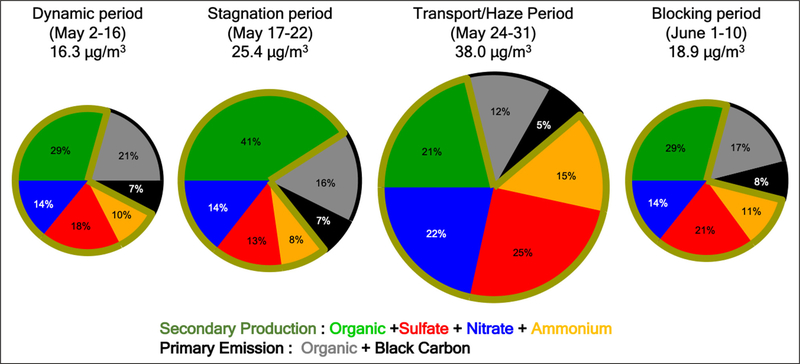
Mean PM_1_ composition observed at the KIST ground site in Seoul for each of the 4 meteorological periods during KORUS-AQ. The size of the pie charts is scaled to the total mean aerosol concentration of the period. The outer black arc on each pie signifies primary aerosols, while the green outline encompasses secondary aerosols. DOI: https://doi.org/10.1525/elementa.424.f8

Differences in aerosol chemical composition for the various meteorological periods of the campaign observed from the DC-8 and at the ground sites in Seoul offer another source of insight into the factors governing PM_2.5_ abundance. The chemical composition measurements here are limited to the PM_1_ size fraction which excludes aerosols in the 1–2.5 μm diameter range that contribute to the PM_2.5_ measurements presented in the preceding sections. Generally, there was little difference between the hourly PM_1_ and PM_2.5_ mass loadings at Olympic Park throughout the campaign (Fig. S4). Larger differences were found during the Transport/Haze period, the implications of which will be discussed further in Section 3.2.4. The mean PM_1_ aerosol composition observed at KIST in Seoul shown in Figure 8 provides an illustration of the differences found during the Dynamic, Stagnant, Transport/Haze, and Blocking periods.

A more quantitative comparison is offered in Tables 1 and 2 by contrasting mass concentrations of each constituent during the four meteorological periods of the campaign to overall means observed at the KIST site from April 14^th^ to June 15^th^. First, little difference in primary concentrations (primary organic aerosol, POA, and black carbon, BC) was found throughout the campaign, suggesting that the dominant processes driving aerosol variability involved secondary production rather than primary emissions. Second, the Transport/Haze period exhibited by far the largest mass concentrations of the major inorganic aerosol ions, exceeding April–June averages by more than a factor of two (ammonium, 116%, sulfate, 116%, and nitrate, 117% greater than the means). By contrast, the mass concentrations of these ions during the Stagnant period were lower than April–June averages by 16%, 26%, and 3%, respectively. Similarly, ALW was calculated to be enhanced by 138% during the Transport/Haze period and reduced by 70% during the Stagnant period compared to the April–June mean. The relationship between the inorganic ions and ALW is discussed in Section 3.2.4. Third, SOA increased both during the Stagnant period and the Transport/Haze period, but the increase was far greater for the former (76%) than the latter (36%). The Transport/Haze period was characterized by overcast and/or partly cloudy conditions (often with fog over the adjacent Yellow Sea) with haze/high-humidity (mean surface RH = 70.2%, Table 1) in a shallow boundary layer, with horizontal surface winds bringing air to the Korean peninsula from across the Yellow Sea. The Stagnant period, however, was clear and dry (mean surface RH = 40.5%, Table 1) under a high-pressure system with limited import of air from external Asian sources. The role of enhanced photochemical production during the Stagnant period with limited removal via transport or deposition resulted in the peak SOA concentrations discussed in more detail in Section 4.1. The differences in Tables 1 and 2 show that meteorology influenced not just the abundance of PM_2.5_, but its composition as well, providing further avenues for investigating the physical and chemical processes behind the variability in PM_2.5_ observed by the AirKorea network.

**Figure 9:**
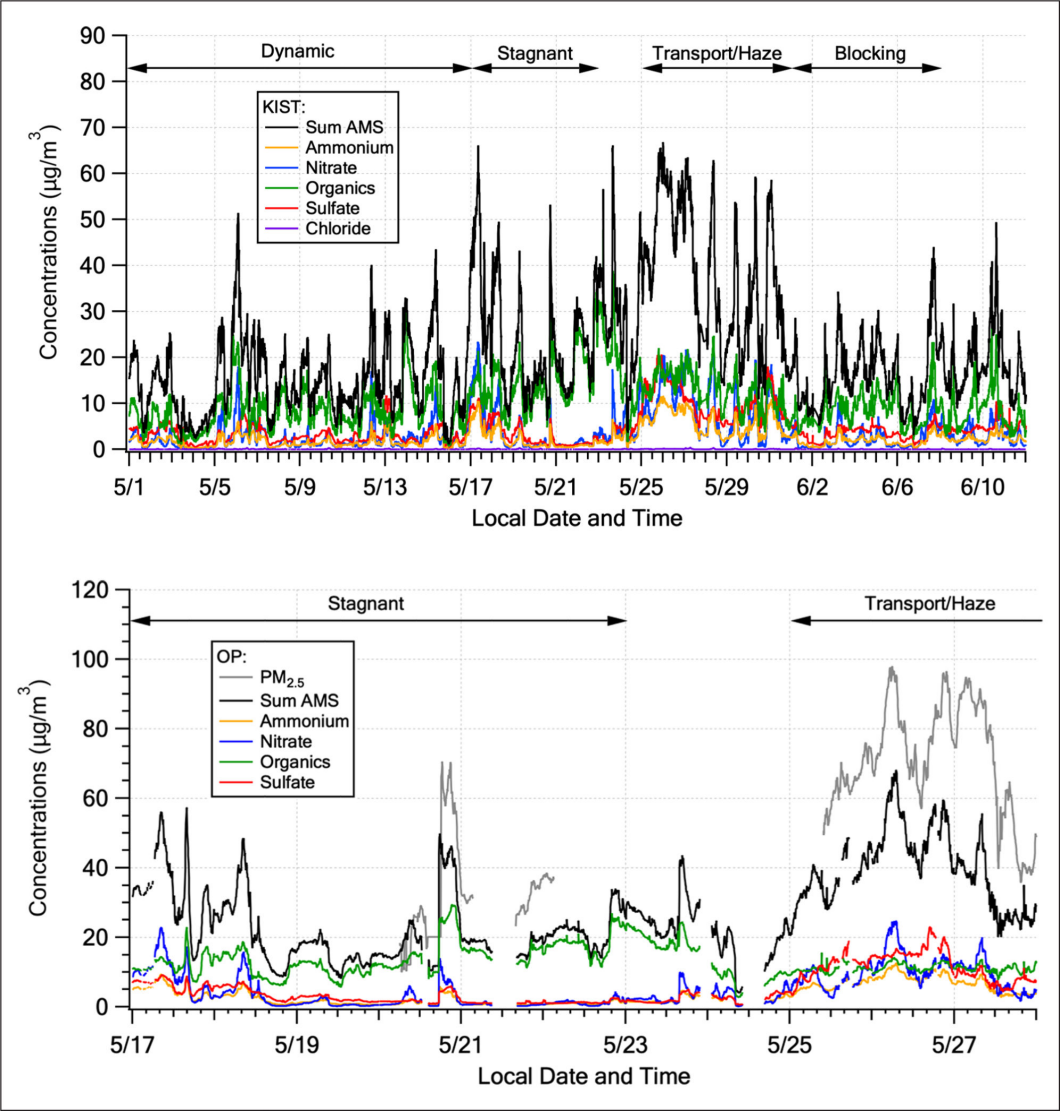
Top panel: campaign time series of KIST AMS data shown with meteorological periods annotated at the top of the figure as in Fig. 2. The sum of the individual constituents (black) is used as a proxy for PM1 aerosol over the entire KORUS-AQ campaign. Bottom panel: Olympic Park AMS Stagnant and 1^st^ half of Transport/Haze periods only, along with 5 min PM2.5 concentrations. DOI: https://doi.org/10.1525/elementa.424.f9

The time series of PM_1_ aerosol composition, along with the sum of the individual components, measured at the KIST site shows the variability of aerosol composition throughout the KORUS-AQ campaign (Figure 9, top panel). There are two key features to note in this figure. First, OA dominated PM_1_ in the latter half of the stagnant period, driving the gradual PM_1_ accumulation over this period. This was a time when PM_2.5_ violated South Korean air quality standards (Peterson et al., 2019). Second, there was less OA during the Transport/Haze period than during the Stagnant period, but that component was accompanied by nearly equal amounts of nitrate, sulfate, and ammonium, such that the sum resulted in the largest concentrations of aerosol during the campaign (Figure 9, top panel, and Table 1). The PM_2.5_ air quality standards were also violated throughout this period (Peterson et al., 2019). In addition to these two features, it is also interesting to note a feature at the start of the Stagnant period (May 17^th^ and 18^th^) that resembles the Transport/Haze period, i.e., equal parts of the inorganic components with the organic component such that the sum resulted in elevated PM_1_. The larger CO/CO_2_ ratios at the start of the Stagnant period (before the decrease to lower values later in the period, Figure 6) suggests that transboundary transport and enhanced inorganic aerosol were important to the large total values of observed PM_1_ both early during the Stagnant period and throughout the Transport/Haze period. Hence, the mean values for the Stagnant period (Figure 8 and Table 1) include contributions from transport and do not solely represent local aerosol under stagnant conditions.

An extensive suite of measurements was made at Olympic Park (the main ground-based observatory in Seoul for KORUS-AQ) including gases (NO_x_, NO_y_, HONO, O_3_, CO, SO_2_, HCHO, and a large number of VOCs) and aerosols (composition, PM_1_, PM_2.5_, and PM_10_). For this reason, further examination of the factors that contributed to the observed PM_2.5_ air quality violations during the campaign will focus on the measurements at Olympic Park, along with DC-8 profiles over that site. The time series of Olympic Park AMS data throughout the campaign (Fig. S5, similar to the top panel of Figure 9) shows that observations between the two sites were typically in good agreement.

As noted above, for most of the campaign the PM_1_ fraction dominated the hourly PM_2.5_ mass loading (Fig. S4). Calculations of mean aerosol volume size distributions from DC-8 observations also show that PM_1_ dominated the aerosol mass for most of the campaign (Fig. S6). However, this was not always the case, especially throughout the Transport/Haze period (Fig. S4). Volume size distributions retrieved from ground-based AERONET remote sensing measurements during the Transport/Haze period were consistent with the DC-8 observations, with both showing enhanced concentrations of large fine mode particles (i.e., enhanced PM_2.5_ compared to PM_1_) (Eck et al., 2020). This raises the question of whether PM_1_ composition measurements may be properly used to infer drivers of PM_2.5_ variability. To assess this question, PM_2.5_ measured at the same temporal resolution as the AMS measurements at Olympic Park were compared (Figure 9 bottom panel). Although there was limited data from this high resolution PM_2.5_ measurement during the Stagnant period, both this and the AMS clearly captured the temporal evolution of the May 20^th^ sea breeze event during which PM_1_ accounted for ~35 μg m^−3^ of the ~45 μg m^−3^ increase in PM_2.5_ (Figure 9 bottom panel). Further, the 5 min PM_2.5_ measurement was in good agreement with the magnitude of the Seoul City mean hourly PM_2.5_ increase (Figures 2 and 5) for this event. Throughout the first half of the Transport/Haze period for which both data sets are available, the variability of PM_2.5_ followed the variability of the sum of Olympic Park AMS constituents (Figure 9 bottom panel). There are some differences in the curves, particularly towards the end of the first half of the Transport/Haze period, but the observed agreement suggests that the PM_1_ AMS measurements reasonably represent PM_2.5_ composition.

#### 3.2.4. Relationships among ALW, RH, and inorganic aerosol mass fraction

The PM_1_ aerosol composition observed using an AMS instrument described in the preceding section excludes the contribution to the aerosol mass provided by water. ALW is a ubiquitous component of aerosol (Nguyen et al., 2016), but it is still challenging to directly measure (Nguyen et al., 2014). Two methods to estimate it involve using either a thermodynamic model (see Section 2.2) (Nguyen et al., 2014, Guo et al., 2015) or a method based on the difference in measured scattering at wet and dry RH (Beyersdorf et al., 2016). Good agreement was found from these two approaches for the KORUS-AQ data set (not shown), but the model approach using ground-based data offered more complete temporal coverage than the measured scattering approach using DC-8 data. Hence, the former ALW estimates will be used in this discussion. Nonetheless, the scattering methodology offers the potential for future measurement-based studies of ALW. The presence of ALW is controlled by RH, aerosol composition, and fog/cloud droplet processes (Hodas et al., 2014; Xue et al., 2014; Pan et al., 2016; Wu et al., 2018) with the inorganic portion of the aerosol being more hygroscopic than the organic (Massoli et al., 2009; Guo et al., 2015; Battaglia et al., 2019). Recent experimental evidence indicates that nitrate plays a dominant role in initiating formation of ALW (Sun et al., 2018).

**Figure 10:**
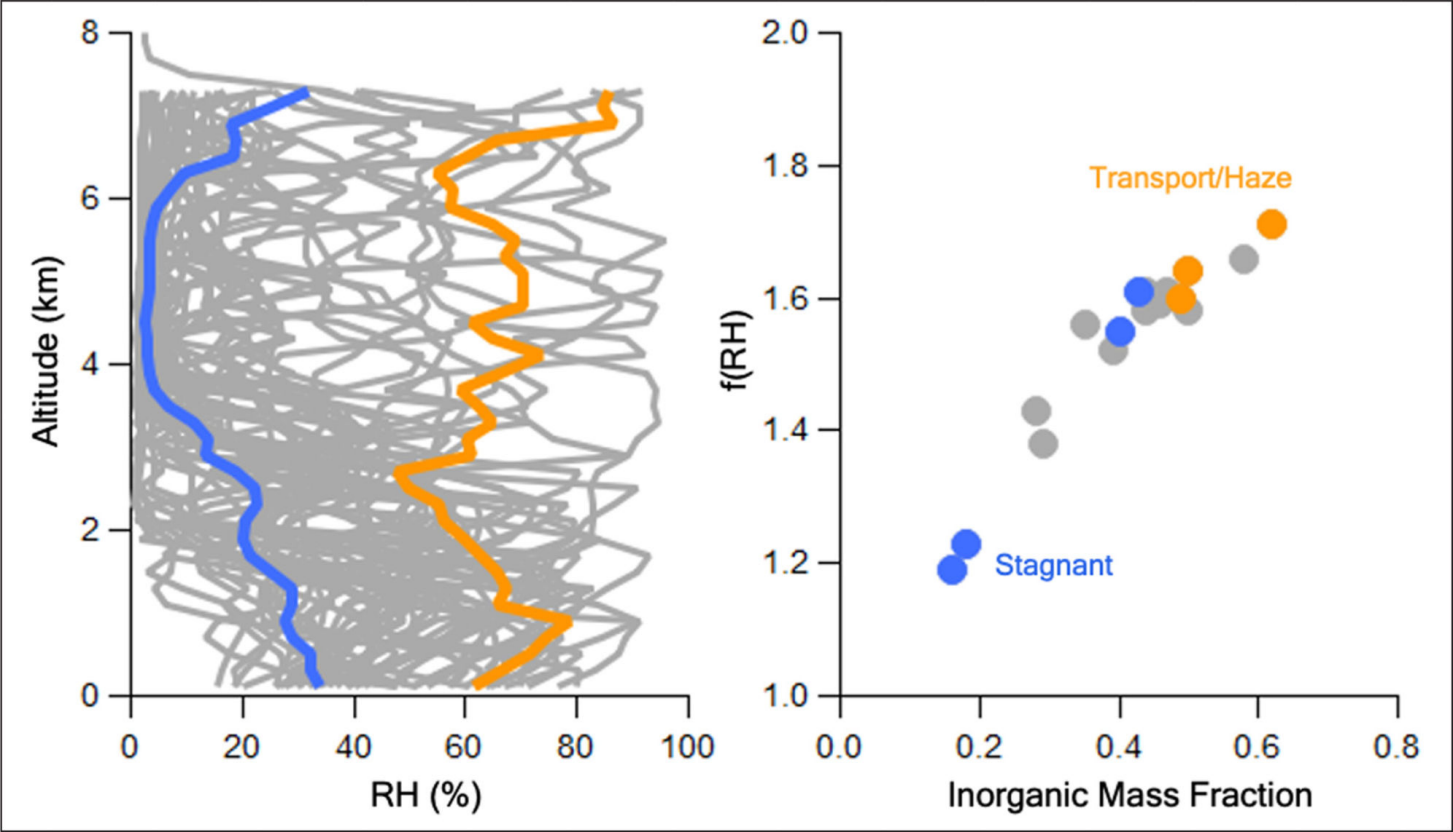
Left panel: All DC-8 vertical profiles of RH (gray curves) over Seoul during KORUS-AQ with mean profiles for Stagnant (blue) and Transport/Haze (orange) periods. Right panel: mean f(RH) and inorganic aerosol mass fraction over Seoul for each flight day (Stagnant flight days in blue, Transport/Haze in orange). The Stagnant mean includes data collected from Seoul spirals on May17^th^, 18^th^, 20^th^, and 22^nd^, while the Transport/Haze mean includes one spiral from May 25^th^ and two on May 26^th^. DOI: https://doi.org/10.1525/elementa.424.f10

Observations from the DC-8 vertical profiles over Seoul illustrate the relationship between RH and the inorganic mass fraction of aerosols. First, mean vertical profiles of RH were starkly different between the Stagnant and Transport/Haze periods with RH ranging from ~60–80% from the ground up to 7 km for the latter period, while it was <40% throughout the entire vertical column during the Stagnant period (Figure 10, left panel). Note, the DC-8 did not fly every day during the campaign. Hence, the Stagnant mean includes data collected from the Seoul spirals on May17^th^, 18^th^, 20^th^, and 22^nd^, while the Transport/Haze mean includes one spiral from May 25^th^ and two on May 26^th^. Although the DC-8 flew on May 30^th^ and 31^st^ (the second half of the Transport/Haze period), those flights were over the Yellow Sea. As a result, the DC-8 data over Seoul was limited to the first half of the Transport/Haze period. Second, the term f(RH) (defined as the ratio of aerosol scattering at 80% RH to that at dry, <40%, RH) is an empirical metric of the inherent ability of aerosols to take up water (Quinn et al., 2005; Beyersdorf et al., 2016), independent of the ambient RH on a particular day. Mean values from the DC-8 vertical profiles over Seoul show f(RH) is a function of the mass fraction of inorganics in the aerosol (Figure 10, right panel). The large inorganic aerosol mass fraction during the Transport/Haze period resulted in values of f(RH) among the highest of the campaign, while the lowest inorganic mass fractions observed at the end of the Stagnant period (May 20^th^ and 22^nd^) resulted in the lowest f(RH) values of the campaign. Note, the two blue dots representing the Stagnant period that fall near the Transport/Haze period dots were from May 17^th^ and 18^th^, the period described in Section 3.2.3 with elevated inorganic mass fractions similar to the Transport/Haze period (Figure 9).

With the varying synoptic conditions, aerosol composition, and f(RH) over the course of the Stagnant period, some care needs to be taken when discussing this period about which set of conditions were prevalent for a specific date within that period. Given the limited transboundary transport during the Stagnant period, it has been convenient to treat this period as representing the baseline of local air quality, which is not entirely appropriate. Even relying on just the latter half of the period to characterize local emissions and secondary production of PM_2.5_ only considers one type of local aerosol production pathway, i.e., photochemical production of SOA. This neglects other local production (and growth) processes involving aqueous and heterogeneous pathways that arise under more humid, hazy conditions.

Ground-based observations of the diurnal variability of T, RH, and UV radiation (Fig. S7) show large differences between the Stagnant and Transport/Haze periods. The reduced UV is consistent with the extensive cloud cover on May 24^th^ and 26^th^ as discussed in Peterson et al. (2019). In addition, fog was present over the Yellow Sea west of the SMA (Peterson et al., 2019; Eck et al., 2020). The low clouds and fog early during the Transport/Haze period may have contributed to the observed shift in the fine volume fraction of aerosols towards larger sizes (i.e. enhanced PM_2.5_ compared to PM_1_) as discussed in Section 3.2.3 (Eck et al., 2020). The fine mode volume retrieved from ground-based AERONET observations was an order of magnitude larger during the Transport/Haze period than the Stagnant period (Eck et al., 2020) and is consistent with aerosol growth arising from the hygroscopicity of inorganic aerosol compounds.

**Figure 11:**
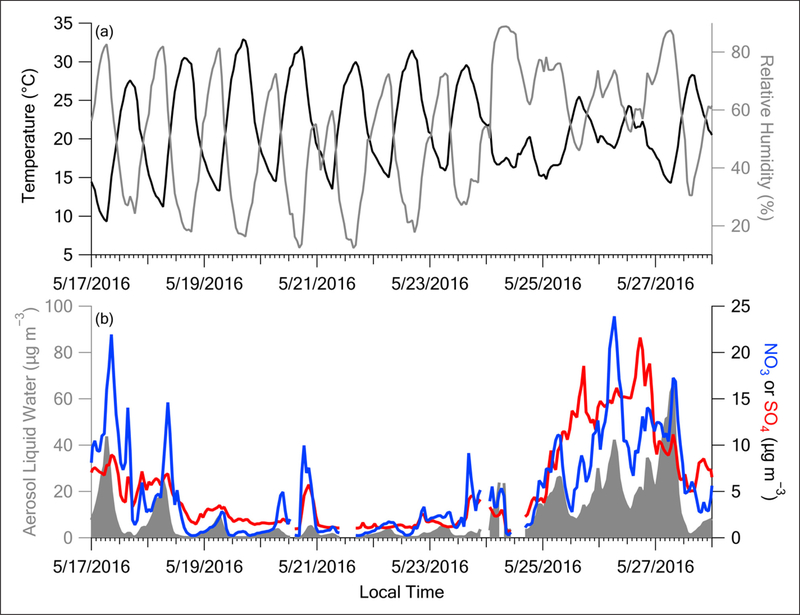
Time series of Olympic Park T and RH (top panel) and ALW with nitrate and sulfate (bottom panel) during the Stagnant and 1^st^ half of Transport/Haze periods. ALW calculated from E-AIM thermodynamic model. DOI: https://doi.org/10.1525/elementa.424.f11

The time series of ALW calculated using the E-AIM thermodynamic model (Section 2.2) covaries with nitrate (Figure 11). High humidity and low temperatures shift the Henry’s Law partitioning from gas-phase NH_3_ and HNO_3_ to aerosol ammonium nitrate (NH_4_NO_3_) (e.g., Wu et al., 2018). Further, the mixture of salts lowers the deliquescence (Wexler and Seinfeld, 1991) and efflorescence (Shaw and Rood, 1990) RH of the aerosol. The hygroscopic nature of particulate nitrate promotes uptake of water into the particle (Guo et al., 2017a). This promotes further partitioning of gas phase NH_3_ and HNO_3_ into the particle phase, causing a positive feedback cycle (Hodas et al., 2014; Xue et al., 2014; Pan et al., 2016; Wu et al., 2018; Ge et al., 2019). This positive feedback creates a tightly coupled system, leading to dramatic increases in ALW and particulate nitrate. Similarly, gas-phase SO_2_ and particulate sulfate participate in this positive feedback mechanism as well. However, once formed sulfate is not semivolatile as is nitrate, hence, the sulfate curve is less tightly coupled to ALW than the nitrate curve (Figure 11).

**Figure 12:**
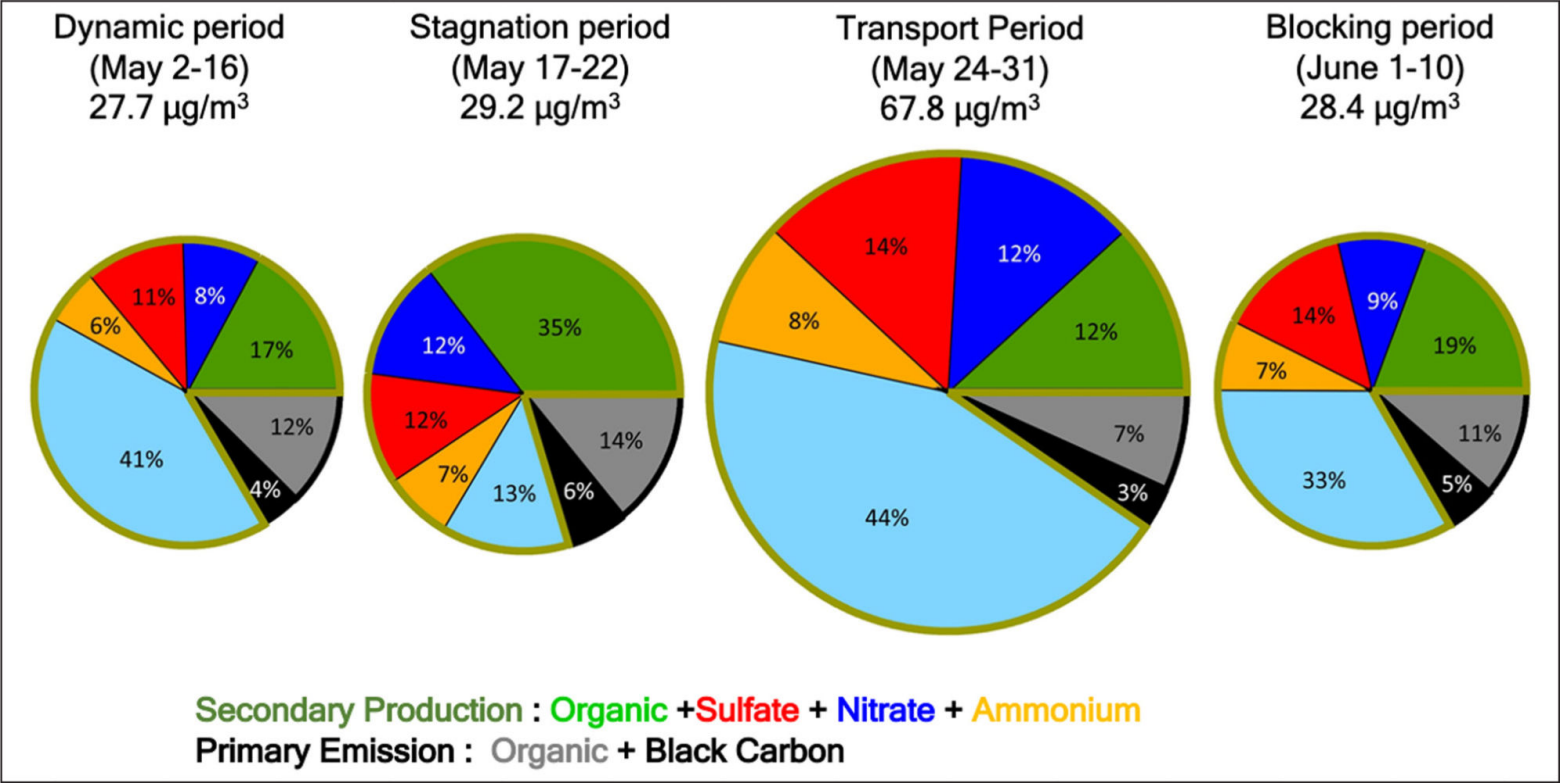
PM_1_ composition as in Figure 8, but with calculated ALW included in the total mass concentrations and pie charts (light blue wedges). ALW calculated from E-AIM thermodynamic model. DOI: https://doi.org/10.1525/elementa.424.f12

The extended period of elevated RH and relatively low T (Figures 11 and S7), with limited diurnal variability of the MLH (Figure 7), in the presence of fog and low clouds early in the Transport/Haze period, contrasts the much drier, warmer, and clear conditions of the Stagnant period with greater diurnal changes in MLH. These differences resulted in a profound difference in ALW for these periods. Adding ALW to the PM_1_ composition shown in Figure 8 reveals that only 13% of the mean PM_1_ mass concentration was apportioned to water during the Stagnant period versus 44% during the Transport/Haze period (Figure 12). ALW is generally considered to be secondary in origin and mostly anthropogenic (Carlton and Turpin, 2013), since the amount of ALW depends on the inorganic aerosol components and their precursors in polluted regions are dominantly anthropogenic (NO_x_ and SO_2_). Hence, control of precursor emissions would be expected to both limit the formation of inorganic aerosol components and the accumulation of ALW, and thereby improve visibility during haze events. The short atmospheric lifetime of NO_x_ makes it unlikely to have been transported into the SMA from abroad. In the analysis of Nault et al. (2018) less than 10% of NO_x_ over Seoul was attributed to transboundary sources. Hence, local emissions account for the rapid production of nitrate via the heterogeneous mechanism described above (see also Section 4.2.1). Production of sulfate may be due to both transported and local precursors (see Section 4.2.2).

## 4. Meteorological influences on the rates of secondary aerosol formation

In the Choi et al. (2019) modeling study the largest discrepancies (30–40 μg m^−3^) between simulated and observed PM_2.5_ were found during the Stagnant and Transport/Haze periods. Given the distinct meteorological conditions and aerosol composition observed between the Stagnant and Transport/Haze periods (Section 3), along with the violations of the South Korean air quality standards due to elevated PM_2.5_ during these periods (Peterson et al., 2019), the role of meteorology in determining rates of secondary aerosol formation deserves more detailed discussion. Several studies have examined the causes of the differences between modeled and observed PM_2.5_ during the Stagnant period finding that the enhanced PM_2.5_ was driven by local SOA production under clear conditions conducive to photochemistry. A brief synopsis of these previous results is provided in Section 4.1. However, the differences found during the Transport/Haze period have received scant attention in the literature thus far, so will be discussed at greater length in Section 4.2. In short, enhanced PM_2.5_ during the Transport/Haze period was driven by heterogeneous secondary inorganic aerosol production under high relative humidity and cloudy conditions (Section 4.2).

### 4.1. Stagnant period PM_2.5_ pollution: Photochemical production of SOA

SOA was an important fraction of the dry PM_1_ mass throughout the campaign, but during the Stagnant period this component dominated the mass (Figure 8). With little difference between PM_1_ and PM_2.5_ mass loadings during the Stagnant period (Fig. S4), the sources and mechanisms controlling SOA were responsible for the violations of South Korean PM_2.5_ air quality standards that occurred late in this period (Peterson et al., 2019). Published studies (Nault et al., 2018; Kim et al. 2018; Choi et al., 2019) from the KORUS-AQ science team have already explored various factors that contributed to the accumulation of SOA during the Stagnant period. Using observations from the DC-8 over the SMA, Nault et al. (2018) found the majority of the SOA produced was from photooxidation of local VOC emissions. These authors also directly quantified the SOA formation potential in air flowing in from China and found it to be much lower than for the local emissions. In particular, VOCs with short lifetimes (<1 day, e.g., xylenes, toluene, trimethylbenzenes, and semi- and intermediate-volatile organic compounds (S/IVOC)) accounted for ~80% of the observed SOA enhancement over SMA (Nault et al., 2018). This is similar to NO_x_, as discussed in Section 3.2.2, in that the short lifetimes indicated minimal influence from transport on the precursors that produced SOA throughout the campaign. However, further research is needed to identify the compounds and sources of S/IVOC, as these compounds were not directly measured during the campaign, and can originate both from fossil fuel and volatile chemical products (e.g., solvents, adhesives, personal care products, pesticides, etc.) (McDonald et al., 2018).

That the increase in SOA was mainly due to rapid photooxidation of locally emitted VOCs was supported by strong correlations with T, solar radiance, VOC concentrations, and other photochemically produced pollutants and products, such as O_3_, formaldehyde, and peroxy acyl nitrate (Kim et al., 2018; Nault et al., 2018). However, fully representing SOA production in chemical transport models is an ongoing challenge due to the wide variety of potential precursors in the VOC class and the myriad photochemical reaction pathways they undergo (Hallquist et al., 2009; Shrivastava et al., 2017). This problem was discussed as it specifically applied to the KORUS-AQ Stagnant period (Choi et al., 2019), with various aspects of the broader challenges explored in numerous prior studies (e.g., Heald et al., 2010; Hodzic et al., 2016, 2020; Woody et al., 2016; Schroder et al., 2018). Here, the GEOS-Chem model underestimated total PM_2.5_ during the Stagnant period in SMA, especially from May 20^th^–May 22^nd^ when PM_2.5_ exceeded the South Korean air quality standard (Choi et al., 2019). Potential causes for the underestimate of SOA in chemical transport models, include emissions (e.g., Woody et al., 2016), transport and dilution of emissions (e.g., Woody et al., 2016), amount of oxidant and thus speed of production (Woody et al., 2016), SOA scheme (Hodzic and Jimenez, 2011; Woody et al., 2016; Ma et al., 2017; Pai et al., 2020), and spatial resolution (Pai et al., 2020). Additional studies to further evaluate these parameters in a chemical transport model with observations collected throughout the Korean peninsula during KORUS-AQ would provide insights to improve understanding of sources and mechanisms controlling SOA over SMA. While the mechanistic specifics of the production of SOA remain under investigation, the relationships revealed in the KORUS-AQ data set make it clear that emission controls of VOCs would reduce locally produced SOA contributing to PM_2.5_. Detailed measurements to improve VOC identification are needed to elucidate key precursors and subsequent photochemical SOA production in order to better constrain emission control targets.

### 4.2. Transport/Haze period PM_2.5_ pollution: Heterogeneous production of secondary inorganic aerosol

Nitrate concentrations are routinely overestimated by models compared to observations (e.g., Thakur et al., 1999; Park, 2004; Fairlie et al., 2010; Heald et al., 2012; Zhang et al., 2012; Bian et al., 2017). Yet, the opposite is the case for sulfate where model concentrations underestimate observations (e.g., Wang et al., 2014; Zheng et al., 2015; Cheng et al., 2016; Li, L., et al., 2018). These opposing errors may be offsetting such that overall inorganic aerosol concentrations may be in reasonable agreement, but the model mechanisms are not properly capturing ambient atmospheric processes. The following sections discuss the KORUS-AQ observations presented in Section 3 for the Transport/Haze period within the context of heterogeneous production of secondary inorganic aerosol. Section 4.2.1 describes the tightly coupled process that links nitrate production to aerosol liquid water uptake. Section 4.2.2 provides a brief overview of the state of the science regarding the rapid production of sulfate aerosols in Asian haze events and how the heterogeneous production of both nitrate and sulfate during such events occurs within a positive feedback mechanism involving the meteorological conditions that promote haze development and persistence.

#### 4.2.1. Nitrate production pathways

Modeling studies generally overestimate nitrate concentrations compared to observations (Bian et al., 2017; Fairlie et al., 2010; Heald et al., 2012; Park, 2004; Thakur et al., 1999; Zhang et al., 2012), and this has largely been attributed to excess HNO_3_ but the cause is unclear. During the Transport/Haze period, nitrate was one of the highest contributors to PM_1_ and the primary driver of ALW that contributes greatly to the low visibility during haze events.

Daytime production of HNO_3_ is photochemical (R1) as the NO_3_ radical (R2 and R3) has a short photolytic lifetime (seconds) (Orlando et al., 1993).

(R1) $$\text{OH}+{\text{NO}}_{2}\to {\text{HNO}}_{3}$$

Reactions R2–R4 are most important at night for HNO_3_ production (e.g., Yun et al., 2018; Kelly et al., 2018; Wang et al., 2018), where M is any third body reactant.

(R2) $${\text{NO}}_{2}+{\text{O}}_{3}\to {\text{NO}}_{3}+{\text{O}}_{2}$$

(R3) $${\text{NO}}_{2}+{\text{NO}}_{3}+M\to {\text{N}}_{2}{\text{O}}_{5}+M$$

(R4) $${\text{N}}_{2}{\text{O}}_{5}+\;\text{aerosol}\;\to 2\times {\text{HNO}}_{3}$$

Other reactions (R5 and R6) compete with R3 for the NO_3_ radical, which can reduce the HNO_3_ produced via R4. Also, R3 is reversible, as N_2_O_5_ can thermally dissociate at warm temperatures (R7).

(R5) $${\text{NO}}_{3}+\text{alkenes}\;\to \text{products}$$

(R6) $${\text{NO}}_{3}+\text{NO}\to 2\times {\text{NO}}_{2}$$

(R7) $${\text{N}}_{2}{\text{O}}_{5}+M\to {\text{NO}}_{2}+{\text{NO}}_{3}+M$$

While there were no ground-based hydroxide (OH) measurements to assess the importance of R1, the DC-8 daytime observations showed that OH in the boundary layer was approximately a factor of 2 lower during the Transport/Haze versus Stagnant period suggesting that less HNO_3_ was produced via R1 during that period. Hence, the enhanced nitrate observed during Transport/Haze c annot be attributed to R1 and subsequent partitioning to aerosol. To assess nighttime chemistry, observations at Olympic Park provide useful constraints on reactions involving the NO_3_ radical (R2–R7). The NO_3_ radical and N_2_O_5_ are most likely not in steady state in the boundary layer, due to continued emissions of NO (R6) and alkenes (R5), and high concentrations of NO_2_ (Brown et al., 2003). However, the production rate of the NO_3_ radical, its relative loss rates via R3 versus R6, and the rate of R4 can be calculated to investigate if R4 can be an important source of HNO_3_, and, thus, aerosol nitrate during the Transport/Haze period.

**Figure 13:**
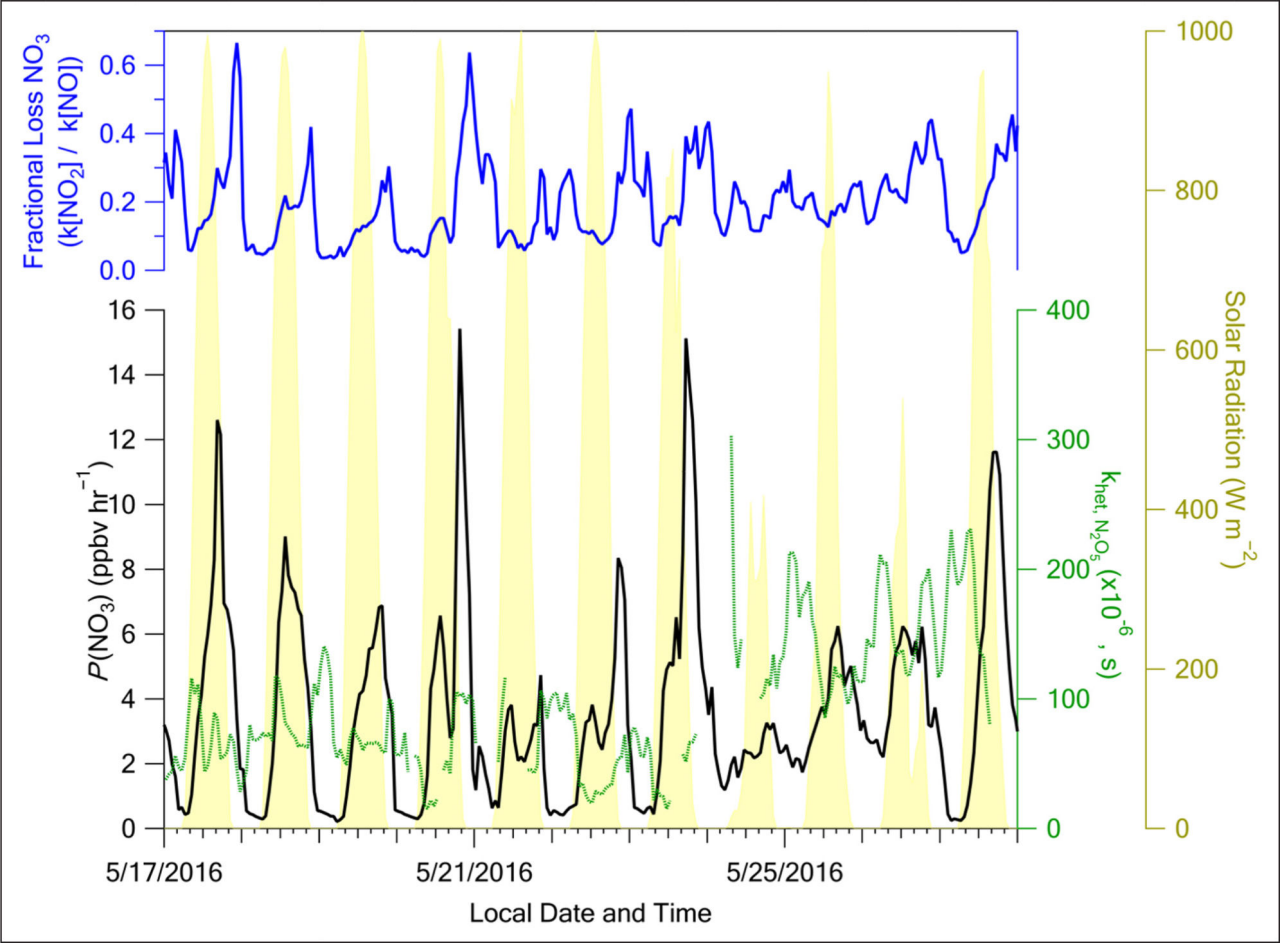
Top blue curve: fraction of the NO_3_ radical lost via its reaction with NO_2_ (reaction R3 in text) versus NO (reaction R6 in text). Lower black curve: production of the NO_3_ radical (P(NO_3_) in ppbv/hr). Lower dotted green curve: heterogeneous reaction rate of N_2_O_5_. Shaded yellow curve: solar radiation (W/m^2^). DOI: https://doi.org/10.1525/elementa.424.f13

The production of NO_3_ radical (*P*(NO_3_), see Section 2.3) is highest as the sun sets during the Stagnant period, decreasing to ≤0.5 ppbv hr^−1^ by 00:00, local time (black curve, Figure 13). This is due to an excess of NO_x_ with respect to O_3_ (Fig. S3), limiting available nighttime O_3_ to continue producing NO_3_ via R2. In contrast, during Transport/Haze, there is sufficient O_3_ at night (Fig. S3) to allow for *P*(NO_3_) = 1–2 ppbv hr^−1^ (black curve, Figure 13). As noted earlier, the greater availability of O_3_ during the Transport/Haze period is likely due to deeper nocturnal mixing during this period reducing the concentrations of NO that would titrate O_3_. Models may have difficulty representing this mechanism due to uncertainties in mixing schemes especially at night (i.e., Zhao et al., 2019). The fractional loss of NO_3_ radical via the competition between R3 (reaction with NO_2_ to form N_2_O_5_) versus R6 (reaction with NO to form NO_2_) remains roughly constant between the Stagnant and Transport/Haze periods (blue curve, Figure 13), with a slight upward trend of R3 becoming more important during the latter period. Finally, the calculated rate constant for R4 was higher (~40% increase) during Transport/Haze than Stagnant (dotted green curve, Figure 13) due to increased surface area and increased ALW during Transport/Haze. These results suggest there is more NO_3_ radical, N_2_O_5_, and hence, HNO_3_ formed from the R2–R4 nocturnal pathway during the Transport/Haze than Stagnant period, leading to more particle nitrate production.

Using the typical N_2_O_5_ lifetime measured in Seoul a year prior to KORUS-AQ (0.2–0.8 hrs, (Brown et al., 2017)), the N_2_O_5_ concentration and in turn particle nitrate production can be estimated with the values shown in Figure 13. Equation 5 (from Brown et al. (2017)) is used to estimate N_2_O_5_ concentration,

(Eq. 5) $$\left[N_{2}O_{5}\right]=\tau_{N_{2}O_{5}}\times P\left(NO_{3}\right)$$

where $\tau_{N_{2}O_{5}}$ is the lifetime of N2O5 (0.2–0.8 hrs) and P(NO_3_) is the production of nitrate radical from Figure 13. This is used to calculate the integrated particle nitrate production each night to compare between the Stagnant and Transport/Haze periods. The integrated production is approximately a factor 3 higher during the Transport/Haze than Stagnant period due to the enhanced nitrate radical production and increased N_2_O_5_ heterogeneous rate constant from the greater ALW and larger surface area. This further supports the impact of local emissions in exacerbating the Transport/Haze PM_2.5_ pollution event. This is being investigated further in ongoing studies.

The overall results of the thermodynamic calculations presented here are consistent with recent studies that found that NO_x_ emissions enhance haze events in South Korea (Jung et al., 2019), the United States (Baasandorj et al., 2017), and China (e.g., Pan et al., 2016; Li, H., et al., 2018; An et al., 2019; Xu et al., 2019). The high levels of ammonia present in Seoul attributed to vehicle traffic (Song et al., 2009; Phan et al., 2013; Link et al., 2017) and regional agriculture (Kim et al., 2011), including rice patties (Das et al., 2009), would be expected to result in NO_x_-limited nitrate formation during our study period. However, models should be assessed as to whether they can reproduce the nitrate production pathways here, particularly during the Transport/Haze period when ozone remains elevated at night.

There are some limitations to this analysis. For example, the impacts of organics on both ALW and N_2_O_5_ heterogeneous uptake were not considered. As shown in prior studies, organics can contribute up to 40% to ALW (Guo et al., 2015; Battaglia et al., 2019). Here, assuming a factor of 2 increase in the ALW only led to an increase in the N_2_O_5_ heterogeneous uptake by 3%; therefore, we do not expect an organic contribution to ALW to impact the rates above. More importantly, organics can inhibit the uptake of N_2_O_5_ to aerosol (e.g., Anttila et al., 2006; Gaston et al., 2014; Liu et al., 2019). During the Stagnant period, this inhibition may have been important as organics contributed a large fraction of the PM_1_ dry mass (41%, Figure 8). Hence, the heterogeneous uptake rate shown in Figure 13 is an upper limit, as is its subsequent contribution to particle nitrate formation. However, during the Transport/Haze period organics contributed only 21% to the dry mass (Figure 8); so, the inhibition of N_2_O_5_ by organics would have been less than that during the Stagnant period. In addition to those two limitations, the dry surface area used here to estimate the N_2_O_5_ uptake was based on average values from the NASA DC-8 (Fig. S6). This results in the estimate here being a lower limit for the uptake rate, since the aerosol surface area would be larger at ambient RH and nighttime surface areas were not observed by the DC-8. All of these factors together suggest the N_2_O_5_ uptake is overestimated for the Stagnant period due to the high organic contribution to aerosol; whereas, it is underestimated during the Transport/Haze period due to the large contribution of ALW, which would increase the surface area. This supports the hypothesis that nitrate production from N_2_O_5_ was more important during the Transport/Haze period.

#### 4.2.2. Haze and enhanced sulfate

In addition to nitrate, sulfate has also been observed to be enhanced in haze events (e.g., Wang et al., 2014; Zheng et al., 2015; Cheng et al., 2016; Kim et al., 2017, 2018; Li, L., et al., 2018, Song et al., 2019). During KORUS-AQ, sulfate concentrations increased along with nitrate concentrations during the Transport/Haze period (Figures 9 and 11). The sulfate precursor, SO_2_, was elevated during the Transport/Haze period (Figure S8) with a dramatic increase in concentration on May 23^rd^ likely due to an approaching cold front that brought transboundary SO_2_ from upwind sources in China. Unlike NO_x_ with a lifetime too short to allow for transport over long distances, in this region SO_2_ has a lifetime of ~9 days (against gas-phase oxidation in the absence of clouds) and ~2 days (in the presence of clouds (Seinfeld and Pandis, 2016)). The faster conversion of SO_2_ to sulfate under the cloudy, foggy, hazy humid conditions of the Transport/Haze period likely arose from both transported and local precursors that contributed to the enhanced local production due to the favorable local meteorological conditions.

Known mechanisms of SO_2_ oxidation (photochemical production via OH, cloudwater production via H_2_O_2_ and O_3_, and O_2_ oxidation catalyzed by transition metal ions (TMI)) cannot account for the observed rapid production of sulfate in haze events (e.g., Wang et al., 2014; Zheng et al., 2015; Cheng et al., 2016; Li, L., et al., 2018). Several recent studies have explored possible alternative pathways not included in most standard models to account for haze-related sulfate production that include heterogeneous reaction pathways involving aqueous aerosol and either NO_2_ (Cheng et al., 2016; Li, L., et al., 2018) or HCHO (Moch et al., 2018; Song et al., 2019), and TMI catalyzed O_2_ oxidation of SO_2_ in haze aerosols (Gankanda et al., 2016; Guo et al., 2017b). A follow-up study will constrain nitrate and sulfate formation mechanisms in the GEOS-Chem chemical transport model using the KORUS-AQ aircraft and ground-based observations and examine whether models can reproduce the enhancements in both species during the Transport/Haze period.

Whichever chemical pathways are found to contribute to the observed increase, it is important to bear in mind that those mechanisms exist within a positive meteorological feedback mechanism as follows: 1) cloud cover and aerosols limit solar radiation in the BL, 2) this restricts diurnal variability of the BL depth compared to clear sky conditions, 3) pollutants accumulate in the humid shallow layer under a temperature inversion, where the operative chemical mechanisms produce more aerosol that act to further stabilize the shallow BL (Qu et al., 2018). The limited diurnal variability of the BL depth and persistent high RH are crucial to the occurrence of haze events (Qu et al., 2018). Hence, the chemical processes and meteorological processes are coupled with a positive feedback dynamic that results in low visibility and poor air quality.

## 5. Recommendations for further study to improve PM_2.5_ air quality

The Rapid Science Synthesis Report (2017) offered two overarching recommendations to improve air quality related to aerosols. First, reduce anthropogenic gas-phase precursors (VOCs, SO_2_, NH_3_, and NO_x_) overall to reduce secondary production of PM_2.5_. This targets the dominant contributions to fine aerosols. Second, strategic reductions of those precursors to focus on mitigation in areas with high population density to achieve the greatest health benefits (e.g., reducing NO_x_ emissions in SMA, targeted SO_2_ controls upwind of SMA) would also be beneficial. Choi et al. (2019) assessed the incremental emission controls that would bring the greatest benefit to the residents of South Korea using modeling based on known mechanisms of aerosol production. They reported that, except for reductions in upwind anthropogenic SO_2_ emissions in China, local controls have the best chance to improve PM_2.5_ air quality. Reductions in anthropogenic NH_3_ and NO_x_ would be the most effective local controls. However, they were careful to note that the recommendations depend strongly on the model simulation of PM_2.5_, hence, further investigations would be very helpful to improve the model to better attribute the sources of PM_2.5_ in South Korea.

Here, we find that under stagnant conditions, rapid photochemical processing of local VOC emissions leads to the observed increase in SOA above South Korean air quality standards. Further, we find that local contributions to PM_2.5_ pollution, enhanced by chemical and meteorological feedbacks, may have been underestimated in previous work that largely attributed PM_2.5_ in the Transport/Haze period to trapped pollution from upwind transport (Kim et al., 2018). The KORUS-AQ aerosol data set described in this work provides evidence for enhanced local production of nitrate and sulfate during the Transport/Haze period via heterogeneous processes. A positive feedback mechanism that couples the production of nitrate and sulfate with the accumulation of ALW was described. Within this feedback mechanism, nitrate production was attributed to local NO_x_ emissions and enhanced nighttime NO_3_ radical chemistry, while sulfate production arises from both local and transported SO_2_ via presently unresolved secondary production pathways. The production of aerosol under the cloudy humid conditions of the Transport/Haze period exacerbates the development and persistence of low visibility haze in a further positive feedback mechanism that couples the aerosol chemical mechanisms to the broader meteorological conditions, including reduced vertical mixing. Hence, restricting the aerosol precursors could help to alleviate the severity and duration of such haze events.

Certain data gaps precluded a quantitative attribution of local versus upwind contributions to PM_2.5_. However, insights gained from the KORUS-AQ campaign suggest that additional targeted monitoring would benefit future scientific assessments, model refinements, and policy development. Additional measurements needed for longer term assessment of PM_2.5_ variability include continuous high quality measurements of CO/CO_2_ (as an indicator of transboundary transport), high-resolution aerosol composition (critical to documenting the interplay between meteorology and gas-to-particle conversion processes), MLH (e.g., using ceilometers to normalize surface PM_2.5_ variations due to mixing), and ALW (calculated from aerosol f(RH) based on humidified and dry aerosol scattering measurements). This augmentation need only be implemented at a few locations in the AirKorea network, but measurements should be co-located to avoid complications in distinguishing local and non-local influences. The need to better constrain contributions to PM_2.5_ that arise from local sources and processes versus upwind sources and precursors that subsequently add to the aerosol burden of South Korea is fundamental to targeting those emissions most likely to improve air quality across South Korea.

### Data accessibility statement

All data used herein are available from the KORUS-AQ data archive via the digital object identifier (doi) 10.5067/Suborbital/KORUS-AQ/DATA01.

## Supplementary Material

Supplementary Material

Sup Data 1

Sup Data 2

Sup Data 3

## Figures and Tables

**Table 1:** PM_1_ aerosol composition measured at KIST, along with meteorological parameters and calculated ALW. Means ± standard deviations with the number of values in the mean in parentheses. DOI:https://doi.org/10.1525/elementa.424.t1

	April–June Mean	Dynamic Period	Stagnant Period	Transport Period	Blocking Period
POA (μg m^−3^)	3.85 ± 3.18 (11605)	3.35 ± 3.25 (2803)	4.17 ± 2.66 (1200)	4.57 ± 3.29 (1478)	3.19 ± 2.26 (1858)
Black Carbon (μg m^−3^)	1.52 ± 0.82 (28758)	1.21 ± 0.70 (7090)	1.74 ± 0.83 (2879)	2.09 ± 0.88 (3639)	1.48 ± 0.81 (4553)
Ammonium (μg m^−3^)	2.56 ± 2.16 (14403)	1.60 ± 1.16 (3562)	2.14 ± 2.28 (1440)	5.54 ± 2.78 (1821)	2.07 ± 1.06 (2276)
Sulfate (μg m^−3^)	4.40 ± 3.26 (14403)	2.99± 1.83 (3562)	3.25 ± 2.72 (1440)	9.50 ± 4.12 (1821)	3.92 ± 1.52 (2276)
Nitrate (μg m^−3^)	3.78 ± 4.20 (14403)	2.30 ± 2.58 (3562)	3.66 ± 4.95 (1440)	8.20 ± 5.86 (1821)	2.70 ± 2.33 (2276)
SOA (μg m^−3^)	5.91 ± 4.63 (11605)	4.78 ± 2.98 (2803)	10.4 ± 6.06 (1200)	8.04 ± 3.69 (1478)	5.51 ± 2.79 (1858)
Total PM_1_ (μg m^−3^)	22.1 ± 13.0 (14403)	16.3 ± 8.80 (3562)	25.4 ± 12.5 (1440)	38.0 ± 15.6 (1821)	18.9 ± 7.89 (2276)
Temperature (°C)	19.7 ± 4.5 (10773)	17.2 ± 5.0 (3562)	22.0 ± 5.0 (1439)	20.1 ± 3.4 (1820)	22.3 ± 3.3 (2275)
Relative Humidity (%)	60.1 ± 21.3 (10773)	66.3 ± 22.4 (3562)	40.5 ± 17.9 (1439)	70.2 ± 16.4 (1820)	61.3 ± 17.2 (2275)
ALW (μg m^−3^)	12.5 ± 21.9 (10773)	11.4 ± 21.6 (3562)	3.8 ± 7.3 (1439)	29.8 ± 30.4 (1820)	9.5 ± 18.3 (2275)

Abreviations: POA = Primary Organic Aerosol, SOA = Secondary Organic Aerosol, PM_1_ = Particulate Matter with aerodynamic diameters <1 μm, ALW = Aerosol Liquid Water.

**Table 2:** Differences in the means shown in Table 1, between those observed during each meteorological period from the campaign as a whole. Bold values highlight the largest differences from the campaign mean that occurred during the Stagnant and Transport/Haze periods. DOI: https://doi.org/10.1525/elementa.424.t2

	Dynamic Period	Stagnant Period	Transport Period	Blocking Period
POA (pg m^−3^)	−0.5	0.3	0.7	−0.7
Black Carbon (pg m^−3^)	−0.3	0.2	0.6	0.0
Ammonium (pg m^−3^)	−1.0	−0.4	**3.0**	−0.5
Sulfate (pg m^−3^)	−1.4	−1.2	**5.1**	−0.5
Nitrate (pg m^−3^)	−1.5	−0.1	**4.4**	−1.1
SOA (pg m^−3^)	−1.1	**4.5**	2.1	−0.4
Total PM_1_ (pg m^−3^)	−5.8	3.3	**15.9**	−3.2
Temperature (°C)	−2.5	2.3	0.4	2.6
Relative Humidity (%)	6.2	**−19.6**	**10.1**	1.2
ALW (pg m^−3^)	−1.1	**−8.7**	**17.3**	−3.0

Abreviations: POA = Primary Organic Aerosol, SOA = Secondary Organic Aerosol, PM_1_ = Particulate Matter with aerodynamic diameters <1 μm, ALW = Aerosol Liquid Water.

## References

[R1] An Z, Huang RJ, Zhang R, Tie X, Li G, Cao J, Zhou W, Shi Z, Han Y, Gu Z and Ji Y 2019 Severe haze in northern China: A synergy of anthropogenic emissions and atmospheric processes. PNAS 116(18): 8657–8666. DOI: 10.1073/pnas.1900125116 30988177 PMC6500134

[R2] Anttila T, Kiendler-Scharr A, Tillman R and Mentel TF. 2006 On the reactive uptake of gaseous compounds by organic-coated aqueous aerosols: Theoretical analysis and application to the heterogeneous hydrolysis of N_2_O_5_. J Phys Chem A 110(35): 10435–10443. DOI: 10.1021/jp062403c 16942049

[R3] Baasandorj M, Hoch SW, Bares R, Lin JC, Brown SS, Millet DB, Martin R, Kelly K, Zarzana KJ, Whiteman CD, Dube WP, Tonnesen G, Jaramillo IC and Sohl J 2017 Coupling between chemical and meteorological processes under persistent cold-air pool conditions: Evolution of wintertime PM_2.5_ pollution events and N2O5 observations in Utah’s Salt Lake Valley. Environ Sci Technol 51(11): 5941–5950. DOI: 10.1021/acs.est.6b06603 28468492

[R4] Battaglia MA Jr., Weber RJ, Nenes A and Hennigan CJ. 2019 Effects of water-soluble organic carbon on aerosol pH. Atmos Chem Phys 19: 14,607–14,620. DOI: 10.5194/acp-19-14607-2019

[R5] Bertram TH and Thornton JA. 2009 Toward a general parameterization of N_2_O_5_ reactivity on aqueous particles: the competing effects of particle liquid water, nitrate and chloride. Atmos Chem Phys 9: 8351–8363. DOI: 10.5194/acp-9-8351-2009

[R6] Beyersdorf AJ, Ziemba LD, Chen G, Corr CA, Crawford JH, Diskin GS, Moore RH, Thornhill KL, Winstead EL and Anderson BE. 2016 The impacts of aerosol loading, composition, and water uptake on aerosol extinction variability in the Baltimore–Washington, D.C. region. Atmos Chem Phys 16: 1003–1015. DOI: 10.5194/acp-16-1003-2016

[R7] Bian H, Chin M, Hauglustaine DA, Schulz M, Myhre G, Bauer SE, Lund MT, Karydis VA, Kucsera TL, Pan X, Pozzer A, Skeie RB, Steenrod SD, Sudo K, Tsigaridis K, Tsimpidi AP and Tsyro SG. 2017 Investigation of global particulate nitrate from the AeroCom phase III experiment. Atmos. Chem. Phys 17(21): 12911–12940. DOI: 10.5194/acp-17-12911-2017

[R8] Brown SS, An H, Lee M, Park J-H, Lee S-D, Fibiger DL, McDuffie EE, Dubé WP, Wagner NL and Min K-E. 2017 Cavity enhanced spectroscopy for measurement of nitrogen oxides in the Anthropocene: Results from the Seoul tower during MAPS 2015. Faraday Discuss 200: 529–557. DOI: 10.1039/C7FD00001D 28580969

[R9] Brown SS, Stark H and Ravishankara AR. 2003 Applicability of the steady state approximation to the interpretation of atmospheric observations of NO_3_ and N_2_O_5_. J Geophys Res 108(D17). DOI: 10.1029/2003JD003407

[R10] Burkholder JB, Sander SP, Abbatt J, Barker JR, Huie RE, Kolb CE, Kurylo MJ, Orkin VL, Wil-mouth DM and Wine PH. 2015 Chemical Kinetics and Photochemical Data for Use in Atmospheric Studies, Evaluation No. 18. Pasadena: JPL Publication 15–10. Jet Propulsion Laboratory. Available at http://jpldataeval.jpl.nasa.gov Last accessed 1 April 2020.

[R11] Carlton AG and Turpin BJ. 2013 Particle partitioning potential of organic compounds is highest in the Eastern US and driven by anthropogenic water. Atmos Chem Phys 13: 10,203–10,214. DOI: 10.5194/acp-13-10203-2013

[R12] Cheng Y, Zheng G, Wei C, Mu Q, Zheng B, Wang Z, Gao M, Zhang Q, He K, Carmichael G, Pöschl U and Su H 2016 Reactive nitrogen chemistry in aerosol water as a source of sulfate during haze events in China. Sci Advances 2: e1601530 DOI: 10.1126/sciadv.1601530 28028539 PMC5176349

[R13] Choi J, Park RJ, Lee HM, Lee S, Jo DS, Jeong JI, Henze DK, Woo JH, Ban SJ, Lee MD, Lim CS, Park MK, Shin HJ, Cho S, Peterson D and Song CK. 2019 Impacts of local vs. trans-boundary emissions from different sectors on PM_2.5_ exposure in South Korea during the KORUS-AQ campaign. Atmos Environ 203: 196–205. DOI: 10.1016/j.atmosenv.2019.02.008

[R14] Clegg SL, Brimblecombe P and Wexler AS. 1998 A thermodynamic model of the system H^+^ – NH4+ – SO42− – NO3– – H_2_O at tropospheric temperatures. J Phys Chem A 102(12): 2137–2154. DOI: 10.1021/jp973042r

[R15] Crawford JH, Ahn J-Y, Al-Saadi J, Chang L, Emmons L, Kim J, Lee G, Park J-H, Park R, Woo JHand Lefer B. 2020 The Korea-United States Air Quality (KORUS-AQ) Field Study. Elem Sci Anth Manuscript in preparation. 10.1525/elementa.2020.00163 PMC8675105 34926709

[R16] Das P, Sa J-H, Kim Kand Jeon E-C. 2009 Effect of fertilizer application on ammonia emission and concentration levels of ammonium, nitrate, and nitrite ions in a rice field. Environ Monit Assess 154: 275–282. DOI: 10.1007/s10661-008-0395-2 18663592

[R17] DeCarlo PF, Kimmel JR, Trimborn A, Northway MJ, Jayne JT, Aiken AC, Gonin M, Fuhrer K, Horvath T, Docherty KS, Worsnop DR and Jimenez JL. 2006 Field-deployable, high-resolution, time-of-flight aerosol mass spectrometer. Anal Chem 78: 8281–8289. DOI: 10.1021/ac061249n 17165817

[R18] Diskin GS, Podolske JR, Sachse GW and Slate TA. 2002 Open-path airborne tunable diode laser hygrometer. Proceedings of SPIE 196: 4817 DOI: 10.1117/12.453736

[R19] Eck TF, Holben BN, Kim J, Beyersdorf AJ, Choi M, Lee S, Koo JH, Giles DM, Schafer JS, Sinyuk A, Peterson DA, Reid JS, Arola A, Slutsker I, Smirnov A, Sorokin M, Kraft J, Crawford JH, Anderson BE, Thornhill KL, Diskin G, Kim SW and Park S 2020 Influence of cloud, fog, and high relative humidity during pollution transport events in South Korea: aerosol properties and PM_2.5_ variability. Atmos Environ 232: 117530 DOI: 10.1016/j.atmosenv.2020.117530

[R20] Fairlie TD, Jacob DJ, Dibb JE, Alexander B, Avery MA, van Donkelaar A and Zhang L 2010 Impact of mineral dust on nitrate, sulfate, and ozone in transpacific Asian pollution plumes. Atmos Chem Phys 10(8): 3999–4012. DOI: 10.5194/acp-10-3999-2010

[R21] Faloona IC, Tan D, Lesher RL, Hazen NL, Frame CL, Simpas JB, Harder H, Martinez M, Di Carlo P, Ren X and Brune WH. 2004 A laser-induced fluorescence instrument for detecting tropospheric OH and HO_2_: Characteristics and calibration. J Atmos Chem 47: 139–167. DOI: 10.1023/B:JOCH.0000021036.53185.0e

[R22] Friese E and Ebel A 2010 Temperature dependent thermodynamic model of the system H^+^ – NH4+ – Na^+^ – SO42− – NO3− – Cl^−^ – H_2_O. J Phys Chem A 114(43): 11595–11631. DOI: 10.1021/jp101041j 21504090

[R23] Gankanda A, Coddens EM, Zhang Y, Cwiertny DM and Grassian VH. 2016 Sulfate formation catalyzed by coal fly ash, mineral dust and iron (III) oxide: Variable influence of temperature and light. Environ Sci: Processes Impacts 18: 1484–1491. DOI: 10.1039/C6EM00430J 27796391

[R24] Gaston CJ, Thornton JA and Ng NL. 2014 Reactive uptake of N_2_O_5_ to internally mixed inorganic and organic particles: the role of organic carbon oxidation state and inferred organic phase separations. Atmos Chem Phys 14: 5693–5707. DOI: 10.5194/acp-14-5693-2014

[R25] Ge B, Xu X, Ma Z, Pan X, Wang Z, Lin W, Ouyang B, Xu D, Lee J, Zheng M, Ji D, Sun Y, Dong H, Squires FA, Fu P and Wang Z 2019 Role of ammonia on the feedback between AWC and inorganic aerosol formation during heavy pollution in NCP. Earth and Space Science 6: 1675–1693. DOI: 10.1029/2019EA000799

[R26] Guo H, Liu J, Froyd KD, Roberts JM, Veres PR, Hayes PL, Jimenez JL, Nenes A and Weber RJ. 2017a Fine particle pH and gas–particle phase partitioning of inorganic species in Pasadena, California, during the 2010 CalNex campaign. Atmos Chem Phys 17: 5703–5719. DOI: 10.5194/acp-17-5703-2017

[R27] Guo H, Weber RJ and Nenes A 2017b High levels of ammonia do not raise fine particle pH sufficiently to yield nitrogen oxide-dominated sulfate production. Scientific Reports 7: 12109 DOI: 10.1038/s41598-017-11704-0 28935864 PMC5608889

[R28] Guo H, Xu L, Bougiatioti A, Cerully KM, Capps SL, Hite JR Jr., Carlton AG, Lee SH, Bergin MH, Ng NL, Nenes A and Weber RJ. 2015 Fine-particle water and pH in the southeastern United States, Atmos Chem Phys 15: 5211–5228. DOI: 10.5194/acp-15-5211-2015

[R29] Halliday HS, DiGangi JP, Choi Y, Diskin GS, Pusede SE, Rana M, Nowak JB, Knote C, Ren X, He H, Dickerson RR and Li Z 2019 Using short-term CO/CO_2_ ratios to assess air mass differences over the Korean Peninsula during KORUS-AQ. J Geophys Res: Atmos 124: 10,951–10,972. DOI: 10.1029/2018JD029697

[R30] Hallquist M, Wenger JC, Baltensperger U, Rudich Y, Simpson D, Claeys M, Dommen J, D onahue NM, George C, Goldstein AH, Hamilton JF, Herrmann H, Hoffmann T, Iinuma Y, Jang M, Jenkin ME, Jimenez JL, Kiendler-Scharr A, Maenhaut W, McFiggans G, Mentel ThF, Monod A, Prévôt ASH, Seinfeld JH, Surratt JD, Szmigielski R and Wildt J. 2009 The formation, properties and impact of secondary organic aerosol: current and emerging issues. Atmos Chem Phys 9: 5155–5236. DOI: 10.5194/acp-9-5155-2009

[R31] He H, Ren X, Li Z and Dickerson RR. 2017 Evaluation of pollutant emissions in North China Plain using aircraft measurements from the Air Chemistry Research In Asia (ARIAs) campaign. American Geophysical Union, Fall Meeting 2017, abstract #A13F-2131. https://ui.adsabs.harvard.edu/abs/2017AGUFM.A13F2131H/abstract.

[R32] Heald CL, Collett JL, Lee T, Benedict KB, Schwandner FM, Li Y, Clarisse L, Hurtmans DR, Van Damme M, Clerbaux C, Coheur P-F, Philip S, Martin RV and Pye HOT. 2012 Atmospheric ammonia and particulate inorganic nitrogen over the United States. Atmos Chem Phys 12(21): 10295–10312. DOI: 10.5194/acp-12-10295-2012

[R33] Heald CL, Kroll JH, Jimenez JL, Docherty KS, DeCarlo PF, Aiken AC, Chen Q, Martin ST, Farmer DK and Artaxo P 2010 A simplified description of the evolution of organic aerosol composition in the atmosphere. Geophys Res Lett 37(8): L08803 DOI: 10.1029/2010GL042737

[R34] Hodas N, Sullivan AP, Skog K, Keutsch FN, Collett JL Jr., Decesari S, Facchini MC, Carlton AG, Laaksonen A and Turpin BJ. 2014 Aerosol liquid water driven by anthropogenic nitrate: Implications for lifetimes of water-soluble organic gases and potential for secondary organic aerosol formation. Environ Sci Technol 48(19): 11,127–11,136. DOI: 10.1021/es5025096 25191968

[R35] Hodzic A and Jimenez JL. 2011 Modeling anthropogenically controlled secondary organic aerosols in megacity: a simplified framework for global and climate models. Geosci Model Dev 4: 901–917. DOI: 10.5194/gmd-4-901-2011

[R36] Hodzic A, Campuzano-Jost P, Bian H, Chin M, Colarco PR, Day DA, Froyd KD, Heinold B, Jo DS, Katich JM, Kodros JK, Nault BA, Pierce JR, Ray E, Schacht J, Schill GP, Schroder JC, Schwarz JP, Sueper DT, Tegen I, Tilmes S, Tsigaridis K, Yu P and Jimenez JL. 2020 Characterization of organic aerosol across the global remote troposphere: A comparison of ATom measurements and global chemistry models. Atmos Chem Phys 20: 4607–4635. DOI: 10.5194/acp-20-4607-2020

[R37] Hodzic A, Kasibhatla PS, Jo DS, Cappa CD, Jimenez JL, Madronich S and Park RJ. 2016 Rethinking the global secondary organic aerosol (SOA) budget: stronger production, faster removal, shorter lifetime. Atmos Chem Phys 16(12): 7917–7941. DOI: 10.5194/acp-16-7917-2016

[R38] Jung J, Ghim YS, Lyu YS, Lim Y, Park J and Sung M 2019 Quantification of regional contributions to fine particles at downwind areas under Asian continental outflows during winter 2014. Atmos Environ 210: 231–240. DOI: 10.1016/j.atmosenv.2019.04.062

[R39] Kelly JT, Parworth CL, Zhang Q, Miller DJ, Sun K, Zondlo MA, Baker KR, Wisthaler A, Nowak JB, Pusede SE, Cohen RC, Weinheimer AJ, Beyersdorf AJ, Tonnesen GS, Bash JO, Valin LC, Crawford JH, Fried A and Walega JG. 2018 Modeling NH_4_NO_3_ over the San Joaquin Valley during the 2013 DISCOVER-AQ campaign. J Geophys Res 123(9): 4727–4745. DOI: 10.1029/2018JD028290 PMC6145493 30245954

[R40] Kim H, Zhang Q and Heo J 2018 Influence of intense secondary aerosol formation and long-range transport on aerosol chemistry and properties in the Seoul Metropolitan Area during spring time: results from KORUS-AQ. Atmos Chem Phys 18: 7149–7168. DOI: 10.5194/acp-18-7149-2018

[R41] Kim H, Zhang Q, Bae GN, Kim JY and Lee SB. 2017 Sources and atmospheric processing of winter aerosols in Seoul, Korea: Insights from real-time measurements using a high-resolution aerosol mass spectrometer. Atmos Chem Phys 17: 2009–2033. DOI: 10.5194/acp-17-2009-2017

[R42] Kim J-S, Bais AL, Kang S, Lee J and Park K. 2011 A semi-continuous measurement of gaseous ammonia and particulate ammonium concentrations in PM_2.5_ in the ambient atmosphere. J Atmos Chem 68: 251–263. DOI: 10.1007/s10874-012-9220-y

[R43] Knepp TN, Szykman JJ, Long R, Duvall RM, Krug J, Beaver M, Cavender K, Kronmiller K, Wheeler M, Delgado R, Hoff R, Berkoff T, Olson E, Clark R, Wolfe D, Van Gilst D and Neil D 2017 Assessment of mixed-layer height estimation from single-wavelength ceilometer profiles. Atmos Meas Tech 10: 3963–3983. DOI: 10.5194/amt-10-3963-2017 29682087 PMC5906814

[R44] Lamb KD, Perring AE, Samset B, Peterson D, Davis S, Anderson BE, Beyersdorf A, Blake DR, Campuzano-Jost P, Corr CA, Diskin GS, Kondo Y, Moteki N, Nault BA, Oh J, Park M, Pusede SE, Simpson IJ, Thornhill KL, Wisthaler A and Schwarz JP. 2018 Estimating source region influences on black carbon abundance, microphysics, and radiative effect observed over South Korea. J Geophys Res: Atmospheres 123: 13,527–13,548. DOI: 10.1029/2018JD029257

[R45] Li H, Zhang Q, Zheng B, Chen C, Wu N, Guo H, Zhang Y, Zheng Y, Li X and He K 2018 Nitrate-driven urban haze pollution during summertime over the North China Plain. Atmos Chem Phys 18: 5293–5306. DOI: 10.5194/acp-18-5293-2018

[R46] Li L, Hoffman MR and Colussi AJ. 2018 Role of nitrogen dioxide in the production of sulfate during Chinese haze-aerosol episodes. Environ Sci Technol 52(5): 2686–2693. DOI: 10.1021/acs.est.7b05222 29378118

[R47] Link MF, Kim J, Park G, Lee T, Park T, Bin Babar Z, Sung K, Kim P, Kang S, Kim JS, Choi Y, Son J, Lim H-J and Farmer DK. 2017 Elevated production of NH_4_NO_3_ from the photochemical processing of vehicle exhaust: Implications for air quality in the Seoul Metropolitan Region. Atmos Environ 156: 95–101. DOI: 10.1016/j.atmosenv.2017.02.031

[R48] Liu L, Wu J, Liu S, Li X, Zhou J, Feng T, Qian Y, Cao J, Tie X and Li G 2019 Effects of organic coating on the nitrate formation by suppressing the N_2_O_5_ heterogeneous hydrolysis: a case study during wintertime in Beijing–Tianjin–Hebei (BTH). Atmos Chem Phys 19: 8189–8207. DOI: 10.5194/acp-19-8189-2019

[R49] Ma PK, Zhao Y, Robinson AL, Worton DR, Goldstein AH, Ortega AM, Jimenez JL, Zotter P, Prévôt ASH, Szidat S and Hayes PL. 2017 Evaluating the impact of new observational constraints on P-S/IVOC emissions, multi-generation oxidation, and chamber wall losses on SOA modeling for Los Angeles, CA. Atmos Chem Phys 17: 9237–9259. DOI: 10.5194/acp-17-9237-2017

[R50] Massoli P, Bates TS, Quinn PK, Lack DA, Baynard T, Lerner BM, Tucker SC, Brioude J, Stohl A and Williams EJ. 2009 Aerosol optical and hygroscopic properties during TexAQS-GoMACCS 2006 and their impact on aerosol direct radiative forcing. J Geophys Res 114: D00F07 DOI: 10.1029/2008JD011604

[R51] Massucci M, Clegg SL and Brimblecombe P 1999 Equilibrium partial pressures, thermodynamic properties of aqueous and solid phases, and Cl_2_ production from aqueous HCl and HNO_3_ and their mixtures. J Phys Chem A 103(21): 4209–4226. DOI: 10.1021/jp9847179

[R52] McDonald BC, de Gouw JA, Gilman JB, Jathar SH, Akherati A, Cappa CD, Jimenez JL, Lee-Taylor J, Hayes PL, McKeen SA, Cui YY, Kim SW, Gentner DR, Isaacman-VanWertz G, Goldstein AH, Harley RA, Frost GJ, Roberts JM, Ryerson TB and Trainer M 2018 Volatile chemical products emerging as largest petrochemical source of urban organic emissions. Science 359(6377): 760–764. DOI: 10.1126/science.aaq0524 29449485

[R53] Metzger S, Steil B, Abdelkader M, Klingmüller K, Xu L, Penner JE, Fountoukis C, Nenes A and Lelieveld J 2016 Aerosol water parameterisation: a single parameter framework. Atmos Chem Phys 16: 7213–7237. DOI: 10.5194/acp-16-7213-2016

[R54] Moch JM, Dovrou E, Mickley LJ, Keutsch FN, Cheng Y, Jacob DJ, Jiang J, Li M, Munger JW, Qiao X and Zhang Q 2018 Contribution of hydroxymethane sulfonate to ambient particulate matter: A potential explanation for high particulate sulfur during severe winter haze in Beijing. Geophys Res Lett 45: 11,969–11,979. DOI: 10.1029/2018GL079309

[R55] Nault BA, Campuzano-Jost P, Day DA, Schroder JC, Anderson B, Beyersdorf AJ, Blake DR, Brune WH, Choi Y, Corr CA, de Gouw JA, Dibb J, DiGangi JP, Diskin GS, Fried A, Huey LG, Kim MJ, Knote CJ, Lamb KD, Lee T, Park T, Pusede SE, Scheuer E, Thornhill KL, Woo JH and Jimenez JL. 2018 Secondary organic aerosol production from local emissions dominates the organic aerosol budget over Seoul, South Korea, during KORUS-AQ. Atmos Chem Phys 18: 17,769–17,800. DOI: 10.5194/acp-18-17769-2018

[R56] Nguyen TKV, Zhang Q, Jimenez JL, Pike M and Carlton AG. 2016 Liquid water: Ubiquitous contributor to aerosol mass. Environ Sci Technol Lett 3(7): 257–263. DOI: 10.1021/acs.estlett.6b00167

[R57] Nguyen TKV, Petters MD, Suda SR, Guo H, Weber RJ and Carlton AG. 2014 Trends in particle-phase liquid water during the Southern Oxidant and Aerosol Study. Atmos Chem Phys 14: 10,911–10,930. DOI: 10.5194/acp-14-10911-2014

[R58] Orlando JJ, Tyndall GS, Moortgat GK and Calvert JG. 1993 Quantum yields for nitrate radical photolysis between 570 and 635 nm. J Phys Chem 97(42): 10,996–11,000. DOI: 10.1021/j100144a017

[R59] Pai SJ, Heald CL, Pierce JR, Farina SC, Marais EA, Jimenez JL, Campuzano-Jost P, Nault BA, Middlebrook AM, Coe H, Shilling JE, Bahreini R, Dingle JH and Vu K 2020 An evaluation of global organic aerosol schemes using airborne observations. Atmos Chem Phys 20: 2637–2665. DOI: 10.5194/acp-20-2637-2020

[R60] Pan Y, Wang Y, Zhang J, Liu Z, Wang L, Tian S, Tang G, Gao W, Ji D, Song T and Wang Y 2016 Redefining the importance of nitrate during haze pollution to help optimize an emission control strategy. Atmos Environ 141: 197–202. DOI: 10.1016/j.atmosenv.2016.06.035

[R61] Park RJ. 2004 Natural and transboundary pollution influences on sulfate-nitrate-ammonium aerosols in the United States: Implications for policy. J Geophys Res 109(D15): D15204 DOI: 10.1029/2003JD004473

[R62] Peterson DA, Hyer EJ, Han SO, Crawford JH, Park RJ, Holz R, Kuehn RE, Eloranta E, Knote C, Jordan CE and Lefer BL. 2019 Meteorology influencing springtime air quality and pollution transport in Korea. Elem Sci Anth 7: 57 DOI: 10.1525/elementa.395

[R63] Phan N-T, Kim K-H, Shon Z-H, Jeon E-C, Jung K and Kim N-J. 2013 Analysis of ammonia variation in the urban atmosphere. Atmos Environ 65: 177–185. DOI: 10.1016/j.atmosenv.2012.10.049

[R64] Qu W, Wang J, Zhang X, Wang Y, Gao S, Zhao C, Sun L, Zhou Y, Wang W, Liu X, Hu H and Huang F 2018 Effect of weakened diurnal evolution of atmospheric boundary layer to air pollution over eastern China associated to aerosol, cloud – ABL feedback. Atmos Environ 185: 168–179. DOI: 10.1016/j.atmosenv.2018.05.014

[R65] Quinn PK, Bates TS, Baynard T, Clarke AD, Onasch TB, Wang W, Rood MJ, Andrews E, Allan J, Carrico CM, Coffman D and Worsnop D 2005 Impact of particulate organic matter on the relative humidity dependence of light scattering: A simplified parameterization. Geophys Res Lett 32: L22809 DOI: 10.1029/2005GL024322

[R66] Rapid Science Synthesis Report. 2017 Available on-line in English (https://espo.nasa.gov/sites/default/files/documents/KORUS-AQ-ENG.pdf) and in Korean (https://espo.nasa.gov/sites/default/files/documents/KORUS-AQ-RSSR.pdf). Last accessed 1 April 2020.

[R67] Sachse GW, Collett JL Jr., Hill GF, Wade LO, Burney LG and Ritter JA. 1991 Airborne tunable diode laser sensor for high-precision concentration and flux measurements of carbon monoxide and methane. In Schiff HI (ed.). 157 DOI: 10.1117/12.46162 Last accessed 1 April 2020.

[R68] Sachse GW, Hill GF, Wade LO and Perry MG. 1987 Fast-response, high-precision carbon monoxide sensor using a tunable diode laser absorption technique. J Geophys Res 92(D2): 2071–2081. DOI: 10.1029/JD092iD02p02071

[R69] Schroder JC, Campuzano-Jost P, Day DA, Shah V, Larson K, Sommers JM, Sullivan AP, Campos T, Reeves JM, Hills A, Hornbrook RS, Blake NJ, Scheuer E, Guo H, Fibiger DL, McDuffie EE, Hayes PL, Weber RJ, Dibb JE, Apel EC, Jaeglé L, Brown SS, Thornton JA and Jimenez JL. 2018 Sources and secondary production of organic aerosols in the northeastern US during WINTER. J Geophys Res: Atmos 123: 7771–7796. DOI: 10.1029/2018JD028475

[R70] Seinfeld JH and Pandis SN. 2016 Atmospheric Chemistry and Physics: From Air Pollution to Climate Change, 3rd ed., Wiley.

[R71] Shaw MA and Rood MJ. 1990 Measurement of the crystallization humidities of ambient aerosol particles. Atmos Environ Part A 24(7): 1837–1841. DOI: 10.1016/0960-1686(90)90516-P

[R72] Shrivastava M, Cappa CD, Fan J, Goldstein AH, Guenther AB, Jimenez JL, Kuang C, Laskin A, Martin ST, Ng NL, Petaja T, Pierce JR, Rasch PJ, Roldin P, Seinfeld JH, Shilling J, Smith JN, Thornton JA, Volkamer R, Wang J, Worsnop DR, Zaveri RA, Zelenyuk A and Zhang Q 2017 Recent advances in understanding secondary organic aerosol: Implications for global climate forcing. Rev Geophys 55: 509–559. DOI: 10.1002/2016RG000540

[R73] Song CH, Park ME, Lee EJ, Lee JH, Lee BK, Lee DS, Kim J, Han JS, Moon KJ and Kondo Y 2009 Possible particulate nitrite formation and its atmospheric implications inferred from the observations in Seoul, Korea. Atmos Environ 43: 2168–2173. DOI: 10.1016/j.atmosenv.2009.01.018

[R74] Song S, Gao M, Xu W, Sun Y, Worsnop DR, Jayne JT, Zhang Y, Zhu L, Li M, Zhou Z, Cheng C, Lv Y, Wang Y, Peng W, Xu X, Lin N, Wang Y, Wang S, Munger JW, Jacob DJ and McElroy MB. 2019 Possible heterogeneous chemistry of hydroxymethanesulfonate (HMS) in northern China winter haze. Atmos Chem Phys 19: 1357–1371. DOI: 10.5194/acp-19-1357-2019

[R75] Sun J, Liu L, Xu L, Wang Y, Wu Z, Hu M, Shi Z, Li Y, Zhang X, Chen J and Li W 2018 Key role of nitrate in phase transitions of urban particles: implications of important reactive surfaces for secondary aerosol formation. J Geophys Res Atmospheres 123(2): 1234–1243, DOI: 10.1002/2017JD027264

[R76] Thakur AN, Singh HB, Mariani P, Chen Y, Wang Y, Jacob DJ, Brasseur G, Müller J-F and Lawrence M. 1999 Distribution of reactive nitrogen species in the remote free troposphere: data and model comparisons. Atmos Environ 33(9): 1403–1422. DOI: 10.1016/S1352-2310(98)00281-7

[R77] Vay SA, Woo JH, Anderson BE, Thornhill KL, Blake DR, Westberg DJ, Kiley CM, Avery MA, Sachse GW, Streets DG, Tsutsumi Y and Nolf SR. 2003 Influence of regional-scale anthropogenic emissions on CO_2_ distributions over the western North Pacific. J Geophys Res 108(D20). DOI: 10.1029/2002JD003094

[R78] Wang H, Lu K, Chen X, Zhu Q, Wu Z, Wu Y and Sun K 2018 Fast particulate nitrate formation via N_2_O_5_ uptake aloft in winter in Beijing. Atmos Chem Phys 18: 10,483–10,495. DOI: 10.5194/acp-18-10483-2018

[R79] Wang Y, Zhang Q, Jiang J, Zhou W, Wang B, He K, Duan F, Zhang Q, Philip S and Xie Y 2014 Enhanced sulfate formation during China’s severe winter haze episode in January 2013 missing from current models. J Geophys Res: Atmos 119: 10,425–10,440. DOI: 10.1002/2013JD021426

[R80] Wexler AS and Clegg SL. 2002 Atmospheric aerosol models for systems including the ions H^+^, NH4+, Na^+^, SO42−, NO3−, Cl^−^, Br^−^ and H_2_O. J Geophys Res 107(D14): 4207 DOI: 10.1029/2001JD000451

[R81] Wexler AS and Seinfeld JH. 1991 Second-generation inorganic aerosol model. Atmos Environ Part A 25(12): 2731–2748. DOI: 10.1016/0960-1686(91)90203-J

[R82] Woody MC, Baker KR, Hayes PL, Jimenez JL, Koo B and Pye HOT. 2016 Understanding sources of organic aerosol during CalNex-2010 using the CMAQ-VBS. Atmos Chem Phys 16: 4081–4100. DOI: 10.5194/acp-16-4081-2016

[R83] Wu Z, Wang Y, Tan T, Zhu Y, Li M, Shang D, Wang H, Lu K, Guo S, Zeng L and Zhang Y 2018 Aerosol liquid water driven by anthropogenic inorganic salts: Implying its key role in haze formation over the North China Plain. Environ Sci Technol Lett 5(3): 160–166. DOI: 10.1021/acs.estlett.8b00021

[R84] Xu Q, Wang S, Jiang J, Bhattarai N, Li X, Chang X, Qiu X, Zheng M, Hua Y and Hao J 2019 Nitrate dominates the chemical composition of PM_2.5_ during haze event in Beijing, China. Sci Total Environ 689: 1293–1303. DOI: 10.1016/j.scitotenv.2019.06.294 31466166

[R85] Xue J, Griffith SM, Yu X, Lau AKH and Yu JZ. 2014 Effect of nitrate and sulfate relative abundance in PM_2.5_ on liquid water content explored through half-hourly observations of inorganic soluble aerosols at a polluted receptor site. Atmos Environ 99: 24–31. DOI: 10.1016/j.atmosenv.2014.09.049

[R86] Yun H, Wang W, Wang T, Xia M, Yu C, Wang Z, Poon SCN, Yue D and Zhou Y 2018 Nitrate formation from heterogeneous uptake of dinitrogen pentoxide during a severe winter haze in southern China. Atmos Chem Phys 18: 17,515–17,527. DOI: 10.5194/acp-18-17515-2018

[R87] Zhang L, Jacob DJ, Knipping EM, Kumar N, Munger JW, Carouge CC, van Donkelaar A, Wang YX and Chen D 2012 Nitrogen deposition to the United States: distribution, sources, and processes. Atmos Chem Phys 12(10): 4539–4554. DOI: 10.5194/acp-12-4539-2012

[R88] Zhao Y, Zhang L, Zhou M, Chen D, Lu X, Tao W, Liu J, Tian H, Ma Y and Fu T-M 2019 Influences of planetary boundary layer mixing parameterization on summertime surface ozone concentration and dry deposition over North China. Atmos Environ 218: 116950 DOI: 10.1016/j.atmosenv.2019.116950

[R89] Zheng GJ, Duan FK, Su H, Ma YL, Cheng Y, Zheng B, Zhang Q, Huang T, Kimoto T, Chang D, Pöschl U, Cheng YF and He KB. 2015 Exploring the severe winter haze in Beijing: the impact of synoptic weather, regional transport, and heterogeneous reactions. Atmos Chem Phys 15: 2969–2983. DOI: 10.5194/acp-15-2969-2015

