# Supplementary material for: Investigation of factors controlling PM2.5 variability across the South Korean Peninsula during KORUS-AQ
Source: Elementa (Wash D C). Author manuscript; Available in PMC 2021 Jan 5. (PMC7784633; doi:10.1525/elementa.424)
Supplement: Supplementary Material [file NIHMS1655454-supplement-Supplementary_Material.pdf]

Supplemental Material

Investigation of factors controlling $PM_{2.5}$ variability across the South Korean Peninsula during KORUS-AQ

Carolyn E. Jordan[1,2,*], James H. Crawford[2], Andreas J. Beyersdorf[2,3], Thomas F. Eck[4,5], Hannah S. Halliday[2,5,a], Benjamin A. Nault[6,7], Lim-Seok Chang[8], JinSoo Park[8], Rokjin Park[9], Gangwoong Lee[10], Hwajin Kim[11,12], Jun-young Ahn[8], Seogju Cho[13], Hye Jung Shin[8], Jae Hong Lee[14], Jinsang Jung[15], Deug-Soo Kim[16], Meehye Lee[17], Taehyoung Lee[10], Andrew Whitehill[18], James Szykman[2,18], Melinda K. Schueneman[6,7], Pedro Campuzano-Jost[6,7], Jose L. Jimenez[6,7], Joshua P. DiGangi[2], Glenn S. Diskin[2], Bruce E. Anderson[2], Richard H. Moore[2], Luke D. Ziemba[2], Marta A. Fenn[2,19], Johnathan W. Hair[2], Ralph E. Kuehn[20], Robert E. Holz[20], Gao Chen[2], Katherine Travis[2,5], Michael Shook[2], David A. Peterson[21], Kara D. Lamb[7,22], Joshua P. Schwarz[22]

[1]National Institute of Aerospace, Hampton, Virginia, United States of America
[2]NASA Langley Research Center, Hampton, Virginia, United States of America
[3]California State University, San Bernardino, California, United States of America
[4]NASA Goddard Space Flight Center, Greenbelt, Maryland, United States of America
[5]Universities Space Research Association, Columbia, Maryland, United States of America
[6]Department of Chemistry, University of Colorado, Boulder, Colorado, United States of America
[7]Cooperative Institute for Research in the Environmental Sciences, University of Colorado, Boulder, Colorado, United States of America
[8]National Institute of Environmental Research, Air Quality Research Division, Incheon, Republic of Korea
[9]School of Earth and Environmental Sciences, Seoul National University, Seoul, Republic of Korea
[10]Hankuk University of Foreign Studies, Seoul, Republic of Korea
[11]Center for Environment, Health and Welfare Research, Korea Institute of Science and Technology, Seoul, Republic of Korea
[12]Department of Energy and Environmental Engineering, University of Science and Technology, Daejeon, Republic of Korea
[13]Seoul Metropolitan Government Research Institute of Public Health and Environment, Gyeonggi-do, Republic of Korea
[14]Harim Engineering, Inc., Seoul, Republic of Korea
[15]Center for Gas Analysis, Korea Research Institute of Standards and Science, Daejeon, Republic of Korea
[16]Kunsan National University, Gunsan, Republic of Korea
[17]Department of Earth and Environmental Sciences, Korea University, Seoul, Republic of Korea
[18]US EPA/Office of Research and Development/Center for Environmental Measurement and Modeling, Research Triangle Park, North Carolina, United States of America
[19]Science Systems and Applications Inc., Hampton, Virginia, United States of America
[20]Space Sciences Engineering Center, University of Wisconsin, Madison, Wisconsin, United States of America
[21]U.S. Naval Research Laboratory, Monterey, California, United States of America
[22]NOAA Earth System Research Laboratory, Chemical Sciences Division, Boulder, Colorado, United States of America
[a]now at EPA, Research Triangle Park, North Carolina, United States of America
[*]corresponding author, Carolyn.Jordan@nasa.gov

List of Contents:

Figure S1. Scatterplot of daily average $PM_{2.5}$ concentrations at individual AirKorea sites versus the daily average of all sites.

Figure S2. Scatterplot of daily average $PM_{2.5}$ concentrations at individual AirKorea sites versus their site averages during the KORUS-AQ study period. Sites in Seoul are colored orange.

Figure S3. Time series of Olympic Park NO and NO_2 (top panel) and O_3 (bottom panel) during the Stagnant and Transport/Haze periods. Measurements made using KENTEK Co., Ltd., (Daejeon, S. Korea, http://kentek.co.kr/xe/en_sub01_01) analyzer models MEZUS-210 (NO_x) and MEZUS-410 (O_3).

Figure S4. Hourly PM_1 and $PM_{2.5}$ concentrations measured with a Thermo Fisher FH62C14 Continuous Ambient Particulate Monitor (Waltham, MA, United States of America) at Olympic Park.

Figure S5. Campaign time series of the sum of both KIST (black) and Olympic Park (light blue) AMS data sets shown with meteorological periods identified (black arrows) along top. The lower sets of curves (Olympic Park data only) use the traditional color scheme for AMS data: nitrate (blue), sulfate (red), ammonium (gold), and organics (green). Note the limited AMS data at Olympic Park during the 2nd part of the Transport/Haze period, hence, the focus on the 1st part of the Transport/Haze period for much of the discussion.

Figure S6. Aerosol volume size distributions ($\mu m^3/cm^3$). Optical particle diameters (calibrated with polystyrene latex spheres) were measured with the LAS on the DC-8 (see section 2.1.4). PM_1 dominates the mean volume size distributions for the campaign overall and for the Stagnant and Transport/Haze periods, in particular. In contrast, May 5th highlights a flight early in the campaign when dust was prevalent such that the coarse fraction dominated the volume size distribution.

Figure S7. Olympic Park UV radiation (mW/cm^2, purple, right axis), T (°C, red, left axis), and RH (%, blue, left axis) during Stagnant and Transport/Haze periods (top black arrows). UV (mW/cm^2) measurements made with Solar Light 501A (Solar Light Company Inc., United States of America) instrument, T and RH instruments described in Section 2.1.3 of the main text.

Figure S8. Olympic Park SO_2 (SO_2 Analyzer model MEZUS-110, KENTEK Co., Ltd., Daejeon, S. Korea) is elevated during Transport/Haze compared to Stagnant. The dramatic increase on the 23rd suggests a frontal passage prior to the start of the Transport/Haze period bringing in an air mass with elevated SO_2 that then decreases somewhat for the remainder of the period. Also note the large brief passage of an SO_2 enhancement on the evening of May 20th, this is the sea breeze event described in Peterson et al. [this issue].

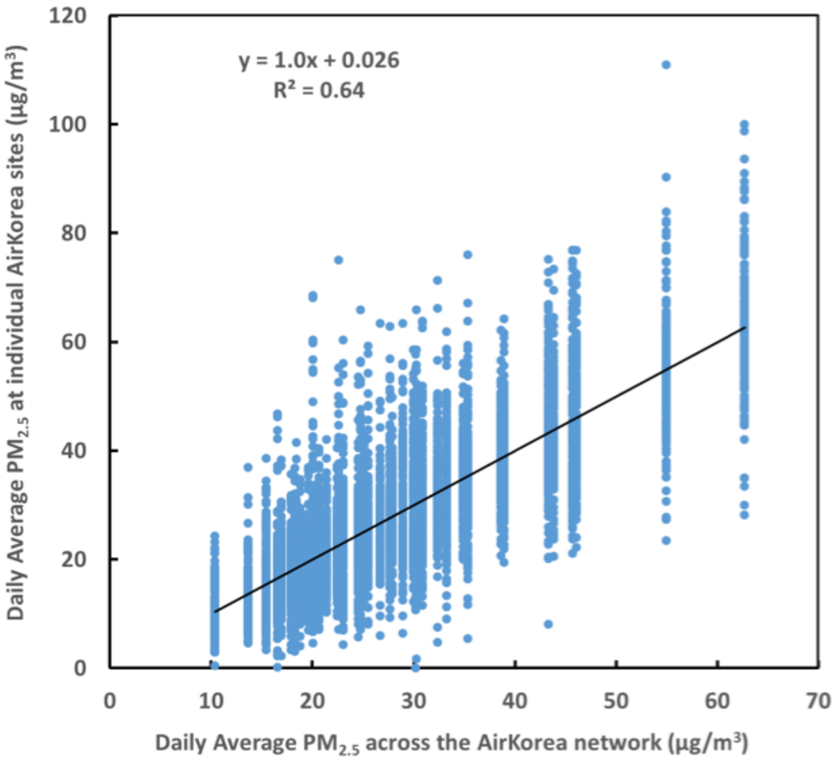

Figure S1. Scatterplot of daily average $PM_{2.5}$ concentrations at individual AirKorea sites versus the daily average of all sites.

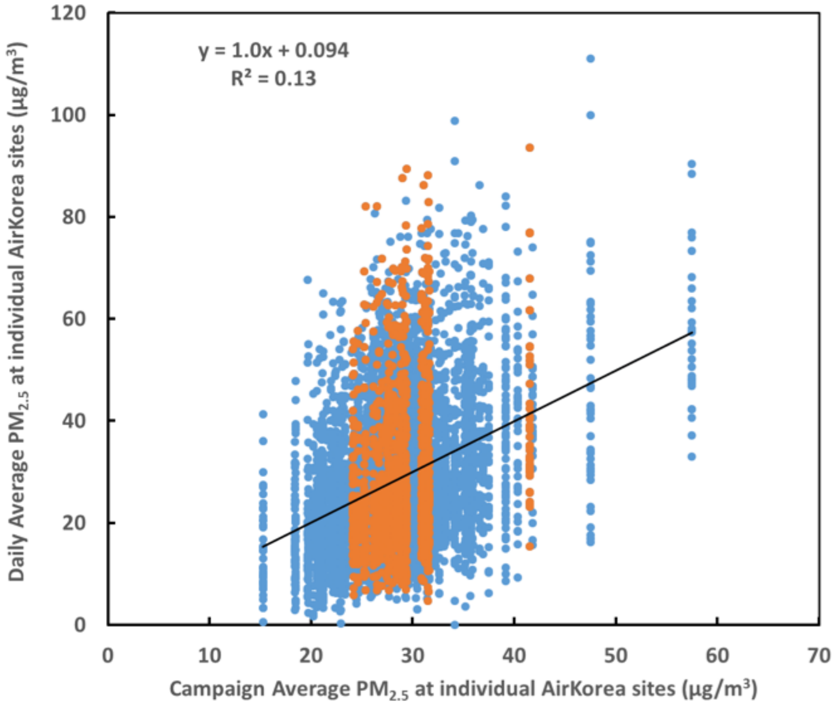

Figure S2. Scatterplot of daily average $PM_{2.5}$ concentrations at individual AirKorea sites versus their site averages during the KORUS-AQ study period. Sites in Seoul are colored orange.

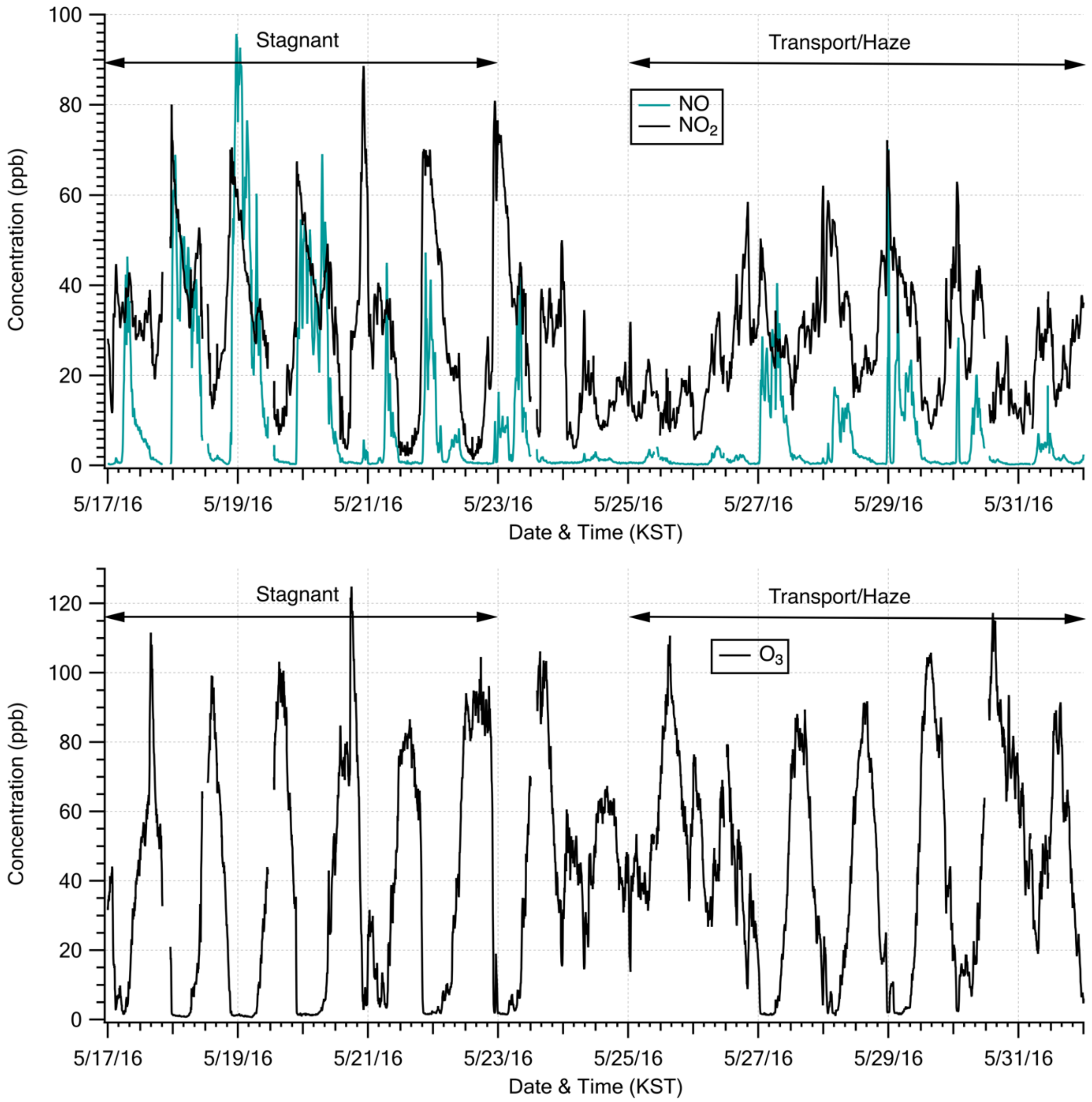

Figure S3. Time series of Olympic Park NO and NO_2 (top panel) and O_3 (bottom panel) during the Stagnant and Transport/Haze periods. Measurements made using KENTEK Co., Ltd., (Daejeon, S. Korea, http://kentek.co.kr/xe/en_sub01_01) analyzer models MEZUS-210 (NO_x) and MEZUS-410 (O_3).

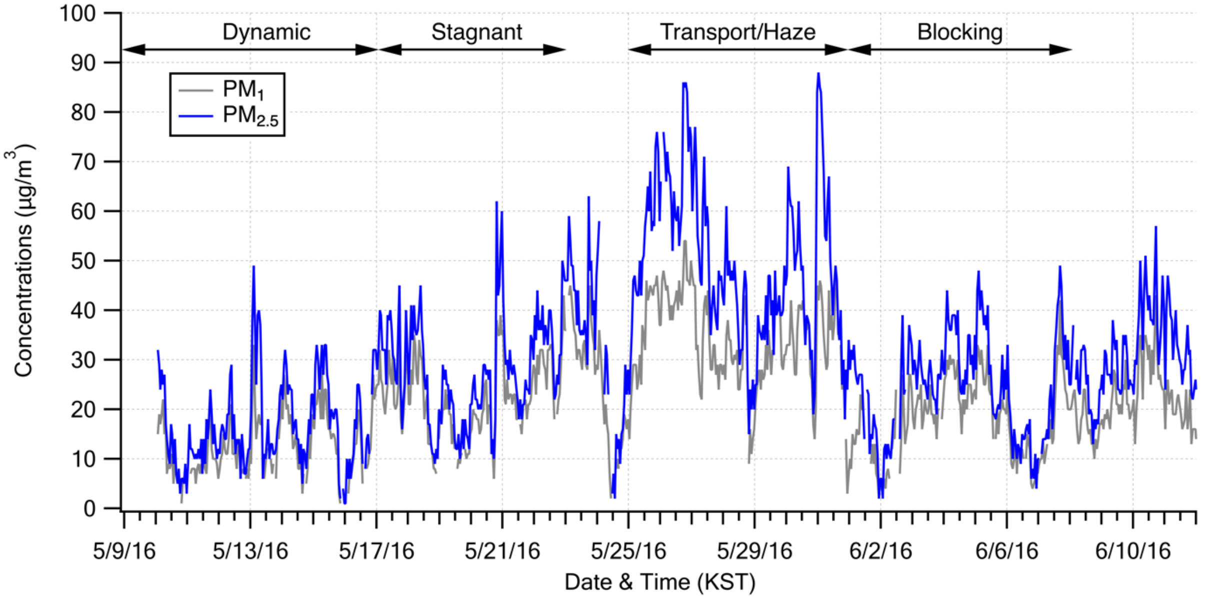

Figure S4. Hourly PM_1 and $PM_{2.5}$ concentrations measured with a Thermo Fisher FH62C14 Continuous Ambient Particulate Monitor (Waltham, MA, United States of America) at Olympic Park.

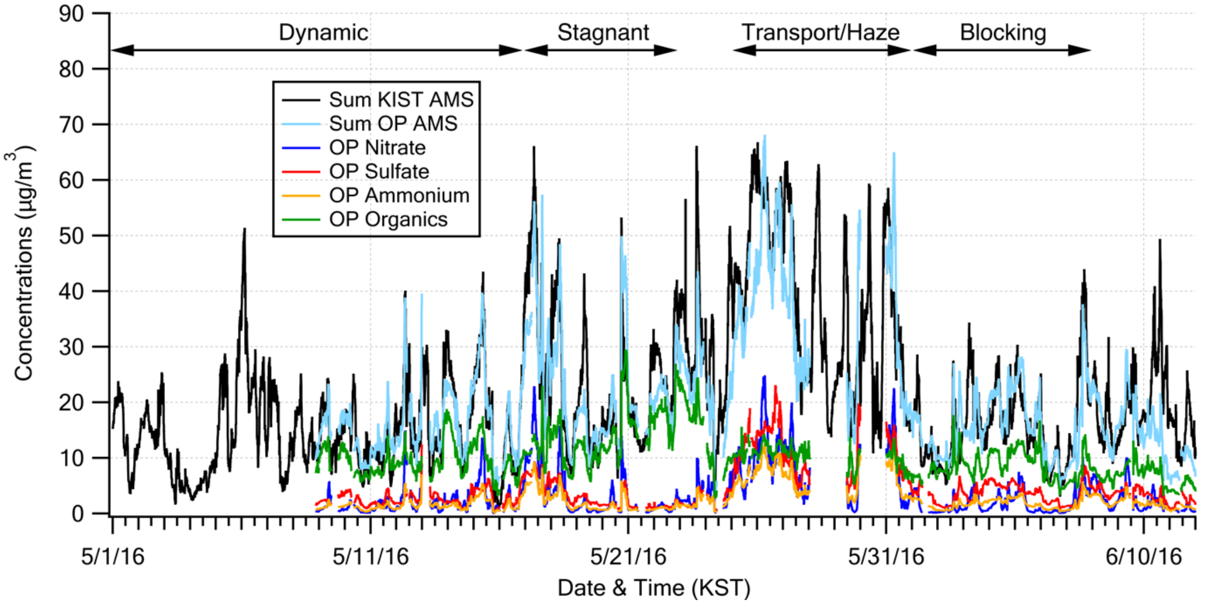

Figure S5. Campaign time series of the sum of both KIST (black) and Olympic Park (light blue) AMS data sets shown with meteorological periods identified (black arrows) along top. The lower sets of curves (Olympic Park data only) use the traditional color scheme for AMS data: nitrate (blue), sulfate (red), ammonium (gold), and organics (green). Note the limited AMS data at Olympic Park during the 2nd part of the Transport/Haze period, hence, the focus on the 1st part of the Transport/Haze period for much of the discussion.

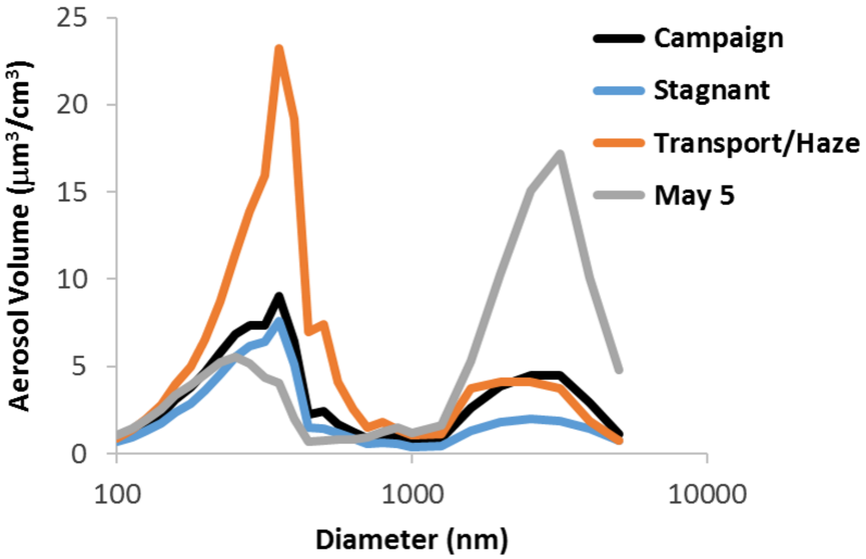

Figure S6. Aerosol volume size distributions ($\mu m^3/cm^3$). Optical particle diameters (calibrated with polystyrene latex spheres) were measured with the LAS on the DC-8 (see section 2.1.4). PM_1 dominates the mean volume size distributions for the campaign overall and for the Stagnant and Transport/Haze periods, in particular. In contrast, May 5th highlights a flight early in the campaign when dust was prevalent such that the coarse fraction dominated the volume size distribution.

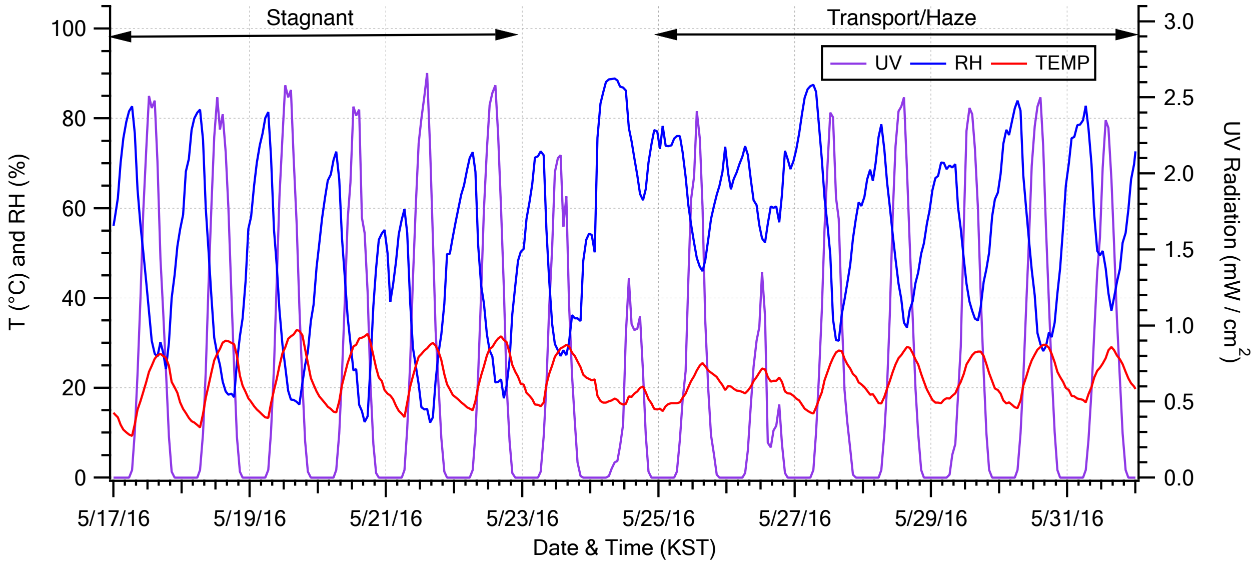

Figure S7. Olympic Park UV radiation (mW/cm^2, purple, right axis), T (°C, red, left axis), and RH (%, blue, left axis) during Stagnant and Transport/Haze periods (top black arrows). UV (mW/cm^2) measurements made with Solar Light 501A (Solar Light Company Inc., United States of America) instrument, T and RH instruments described in Section 2.1.3 of the main text.

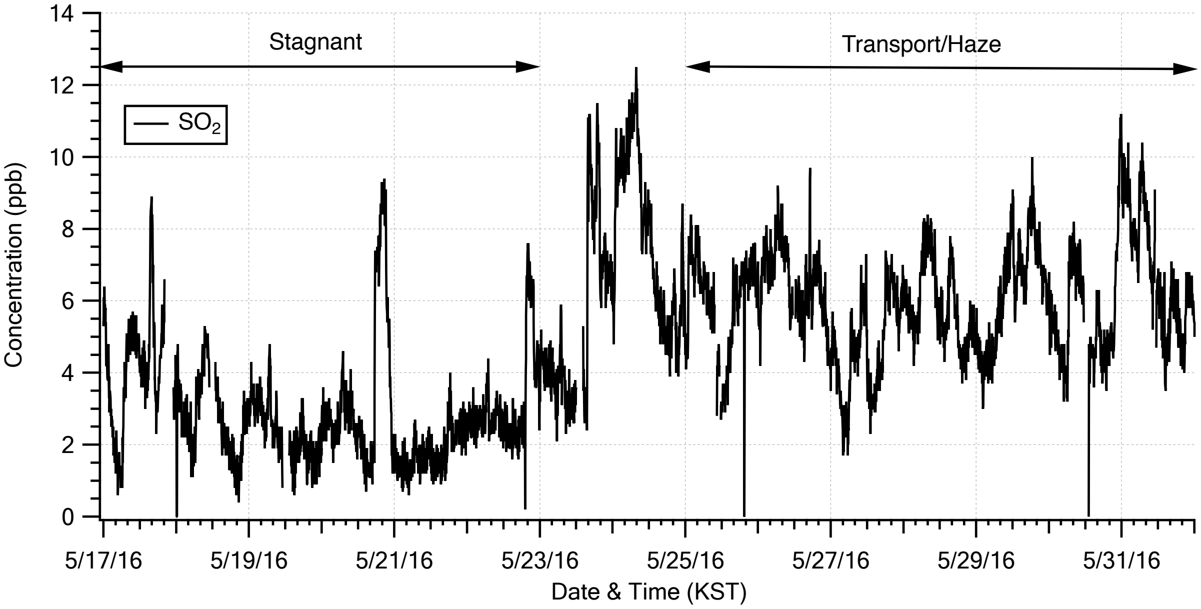

Figure S8. Olympic Park SO_2 (SO_2 Analyzer model MEZUS-110, KENTEK Co., Ltd., Daejeon, S. Korea) is elevated during Transport/Haze compared to Stagnant. The dramatic increase on the 23rd suggests a frontal passage prior to the start of the Transport/Haze period bringing in an air mass with elevated SO_2 that then decreases somewhat for the remainder of the period. Also note the large brief passage of an SO_2 enhancement on the evening of May 20th, this is the sea breeze event described in Peterson et al. [this issue].
